# BUB signaling in cancer and molecular insights into targeted therapies

**DOI:** 10.1038/s42003-026-10976-5

**Published:** 2026-10-16

**Authors:** Bhavika Baweja, Chainsee Saini, Prerna Vats, Rashi Soni, Kritika Jangir, Prachi Patidar, Ashok Kumar, Rajeev Nema

**Affiliations:** 1 Department of Biosciences, Manipal University Jaipur, Dehmi Kalan, Jaipur-Ajmer Expressway, Jaipur, India; 2 Department of Biochemistry, All India Institute of Medical Sciences (AIIMS),Bhopal, Saket Nagar, Bhopal, India

**Keywords:** Cell division, Cancer

## Abstract

The Spindle Assembly Checkpoint (SAC) is vital for proper chromosome segregation during cell division and for maintaining genomic integrity; its disruption can cause aneuploidy and tumors. The BUB gene family ensures SAC fidelity by regulating kinetochore–microtubule attachments and mitotic progression. Dysregulation leads to chromosomal instability (CIN), contributing to cancer progression, heterogeneity, metastasis, and therapy resistance. Beyond mitosis, BUB proteins influence DNA repair, chromatin remodeling, transcription, and immune responses. They interact with cell cycle and genome stability pathways, impacting cancer prognosis and therapy response. Research is ongoing to validate BUB proteins as biomarkers and develop inhibitors, possibly combined with immunotherapies or DNA-damaging agents for resistant tumors. This review  summarizes their functions, highlighting their potential in personalized cancer treatment.

## Abstract (web summary)

This Review examines the diverse roles of BUB proteins in chromosome segregation, cancer progression, and therapy resistance. It highlights emerging therapeutic strategies and future directions for targeting BUB signaling in cancer.

## Background

The Spindle Assembly Checkpoint (SAC) is a critical surveillance mechanism that ensures all chromosomes are correctly attached to the mitotic spindle before cell division proceeds, thereby promoting accurate chromosome segregation, a process essential for maintaining genomic stability^1^. Central to this surveillance system are the BUB (Budding Uninhibited by Benzimidazoles) proteins*—*BUB1, BUB1B (also known as BUBR1), and BUB3, identified as checkpoint factors required for mitotic arrest following spindle perturbation^2,3^. Their deep evolutionary conservation underscores a fundamental role in ensuring mitotic fidelity^3^. At the molecular level, BUB proteins orchestrate checkpoint signaling at kinetochores by coordinating the assembly of the mitotic checkpoint complex (MCC) and inhibiting the anaphase-promoting complex (APC), thereby preventing premature separation of sister chromatids^4^. BUB1 and BUB1B encode serine/threonine kinases with specialized regulatory functions, whereas BUB3 acts as a WD40-repeat scaffold that stabilizes kinetochore-associated signaling complexes^5,6^. Beyond their canonical mitotic roles, BUB proteins participate in diverse non-mitotic pathways, including DNA damage signaling, chromatin organization, transcriptional regulation, replication stress responses, and autophagy, collectively positioning the family as multifunctional regulators that connect cell-cycle progression with genome maintenance, epigenetic remodeling, and tumor adaptation^7–9^.

Dysregulation of BUB expression or activity has profound consequences for tumor initiation and progression^10,11^. The SAC is highly sensitive to BUB dosage, and both loss and overexpression of BUB1 or BUB1B can impair checkpoint robustness, promote chromosome missegregation, and drive chromosomal instability (CIN)^12,13^. CIN and aneuploidy are observed in approximately 90% of solid tumors and nearly 75% of hematological malignancies, highlighting the clinical relevance of SAC components in tumor evolution^14^. Consistently, elevated BUB1 and BUB1B levels correlate with aggressive disease, poor prognosis, therapeutic resistance, and immune evasion across multiple cancer types^15^.

While previous studies have largely emphasized the role of BUB proteins in mitotic checkpoint control, accumulating evidence now supports their broader involvement in oncogenic signaling, tumor plasticity, and therapy response, necessitating an updated integrative framework that bridges mechanistic cell biology with translational oncology. In this review, we examine the structural organization, canonical and non-canonical functions, and regulatory mechanisms of BUB1, BUB1B, and BUB3, with particular emphasis on their integration into oncogenic, immune, and stress-response signaling networks. By integrating recent advances in cell biology, cancer genomics, and translational oncology, this review provides a conceptual framework for understanding how BUB proteins coordinate genome stability, tumor adaptation, and therapeutic response in cancer, while also highlighting the translational and therapeutic relevance of BUB-associated signaling, including emerging biomarker applications and rational combination strategies targeting BUB-dependent vulnerabilities.

## Evolution and structural organization

**Fig. 1:**
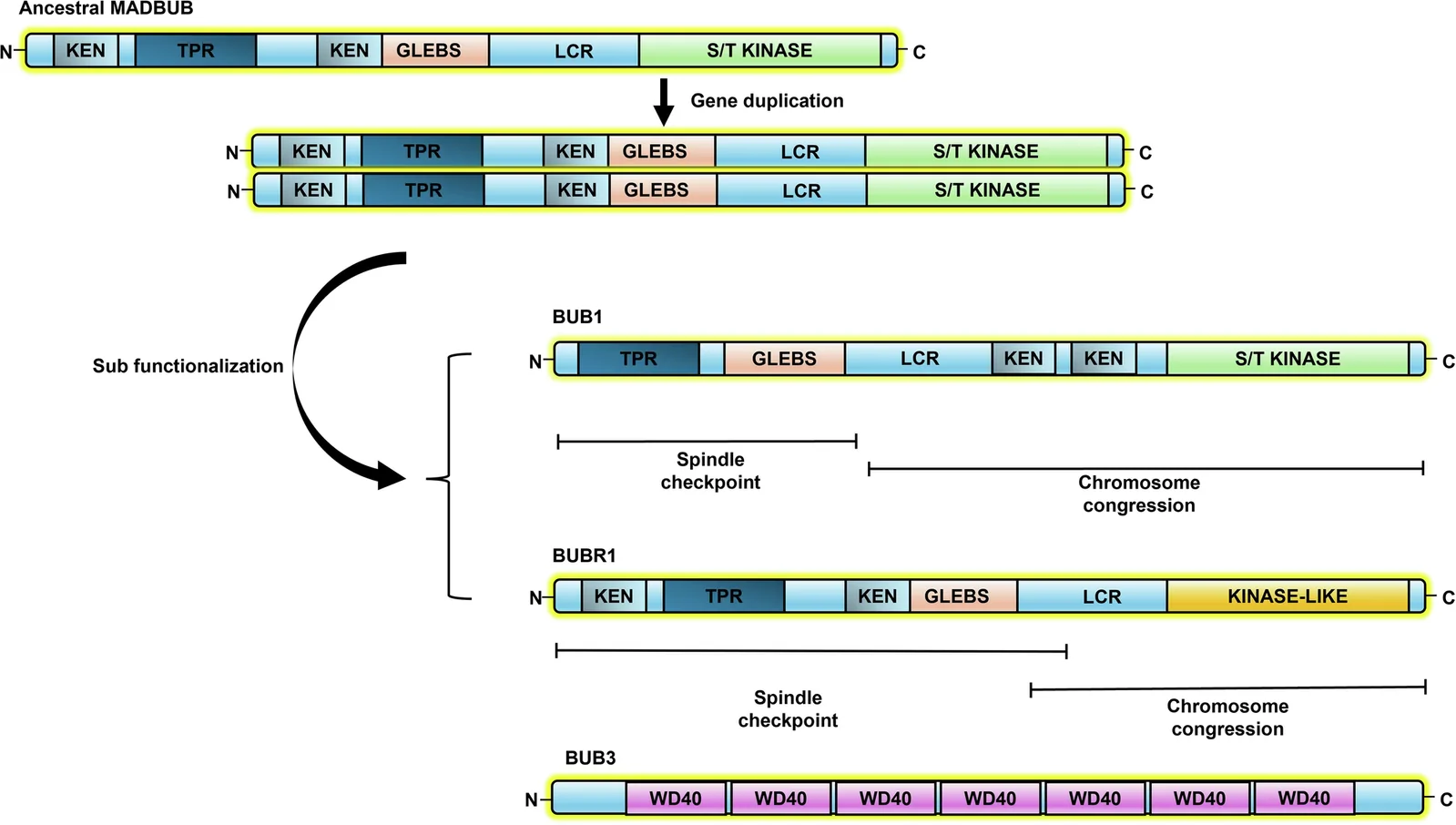
Evolutionary origin and domain organization of the human BUB family proteins. The figure illustrates the ancestral MadBub protein containing KEN motifs, tetratricopeptide repeat (TPR) motifs, a GLEBS motif, and a C-terminal serine/threonine kinase domain is proposed to have undergone gene duplication, giving rise to the human BUB1 and BUB1B proteins. BUB1 retains an active C-terminal serine/threonine kinase domain, whereas BUB1B contains a catalytically inactive pseudokinase domain. The N-terminal region of BUB1 and BUB1B harbors three tandem TPR motifs that mediate kinetochore recruitment through interaction with KNL1, whereas the GLEBS motif binds BUB3. Both proteins contain conserved KEN motifs involved in spindle assembly checkpoint signaling. In contrast, BUB3 consists of seven WD40 repeats that form a seven-bladed β-propeller, providing a scaffold for interaction with the GLEBS motifs of BUB1 and BUB1B.

The BUB gene family encodes evolutionary conserved mitotic checkpoint proteins specialized for dynamic protein-protein interactions and spatial coordination during chromosome segregation^16^. In humans, the BUB family comprises three genes: BUB1 located on chromosome 2q13, BUB1B on chromosome 15q15.1, and BUB3 on chromosome 10q26.13^17–19^. In human cancers, BUB dysregulation is more frequently associated with aberrant expression and dosage imbalance than with recurrent driver mutations^20–22^. Both BUB overexpression and reduced gene dosage can compromise SAC fidelity, promote CIN, and contribute to tumorigenesis, although the biological consequences are highly context-dependent. Advances in structural biology, molecular modeling, and comparative genomics have substantially expanded the understanding of BUB domain architecture and interaction dynamics. As illustrated in Fig. 1, *BUB1, BUB1B*, and *BUB3* exhibit conserved structural frameworks coupled with functional divergence that collectively support kinetochore signaling and SAC regulation. These structural distinctions emerged through evolutionary specialization of ancestral checkpoint proteins, ultimately giving rise to the functionally diverse BUB family observed across modern eukaryotes.

The evolutionary origins of the BUB family are closely linked to the discovery of SAC checkpoint genes in *Saccharomyces cerevisiae*^23,24^. Checkpoint genes including BUB1, BUB2, and BUB3 were initially identified through studies by Hoyt et al., demonstrating that their disruption resulted in premature mitotic exit, leading to the term “Budding Uninhibited by Benzimidazole (BUB)”^23^. Homologs of these genes were subsequently identified in higher eukaryotes, including human BUB1B and vertebrate BUB3, demonstrating deep evolutionary conservation of checkpoint signaling mechanisms^25,26^. Further, phylogenomic analyses suggest that BUB1 and BUB1B evolved through duplication of an ancestral Mitotic Arrest Deficient and BUB Kinase (MADBUB) gene containing both a kinase domain and a Lysine–Glutamic Acid–Asparagine (KEN) box motif involved in checkpoint regulation^27,28^. Following duplication, functional divergence enabled BUB1 to retain catalytic kinase activity, whereas BUB1B evolved predominantly into a pseudokinase scaffold with specialized regulatory functions^5,28^. In contrast, BUB3 diverged as a highly conserved WD40-repeat adaptor protein characterized by a β-propeller architecture that mediates phospho-dependent protein interactions^29,30^. Collectively, these findings highlight the evolutionary diversification generated structurally distinct yet functionally coordinated checkpoint regulators.

Consistent with their evolutionary specialization, structural analyses have revealed that BUB proteins possess modular domain architectures that enable coordinated checkpoint assembly and kinetochore organization^31^. BUB1 contains an N-terminal region harboring a tandem array of three tetratricopeptide repeat (TPR) motifs, a central GLE2-binding sequence (GLEBS) motif, and a catalytically active C-terminal kinase domain^32^. The TPR motifs contribute to kinetochore association, whereas the GLEBS motif mediates interaction with BUB3. Structural studies further indicate that conformational flexibility within the BUB1–BUB3 complex contributes to dynamic checkpoint regulation in response to spindle attachment status^33^. BUB1B shares substantial structural similarity with BUB1, including conserved GLEBS, ABBA, and KEN motifs, but differs in that its C-terminal kinase domain functions as a catalytically inactive pseudokinase that primarily serves as a structural scaffold for SAC signaling rather than mediating protein phosphorylation^28^. Instead, BUB1B contains a kinetochore attachment regulatory domain (KARD) that contributes to checkpoint coordination and attachment stabilization^34^. Structural studies additionally demonstrate that BUB1B serves as a scaffold for large mitotic checkpoint assemblies and outer kinetochore organization through interactions with multiple checkpoint-associated complexes^35^. In parallel, BUB3 functions as a conserved WD40-repeat scaffold protein that facilitates phospho-dependent checkpoint assembly through high-affinity GLEBS interactions with BUB1 and BUB1B^6^. Key structural and functional features of BUB1, BUB1B, and BUB3 are summarized in Supplementary Table 1. Collectively, these structural and evolutionary adaptations establish the BUB family as a highly coordinated regulatory system essential for mitotic checkpoint organization and genome stability across eukaryotic organisms.

## Canonical functions of BUB proteins in spindle assembly checkpoint

**Fig. 2:**
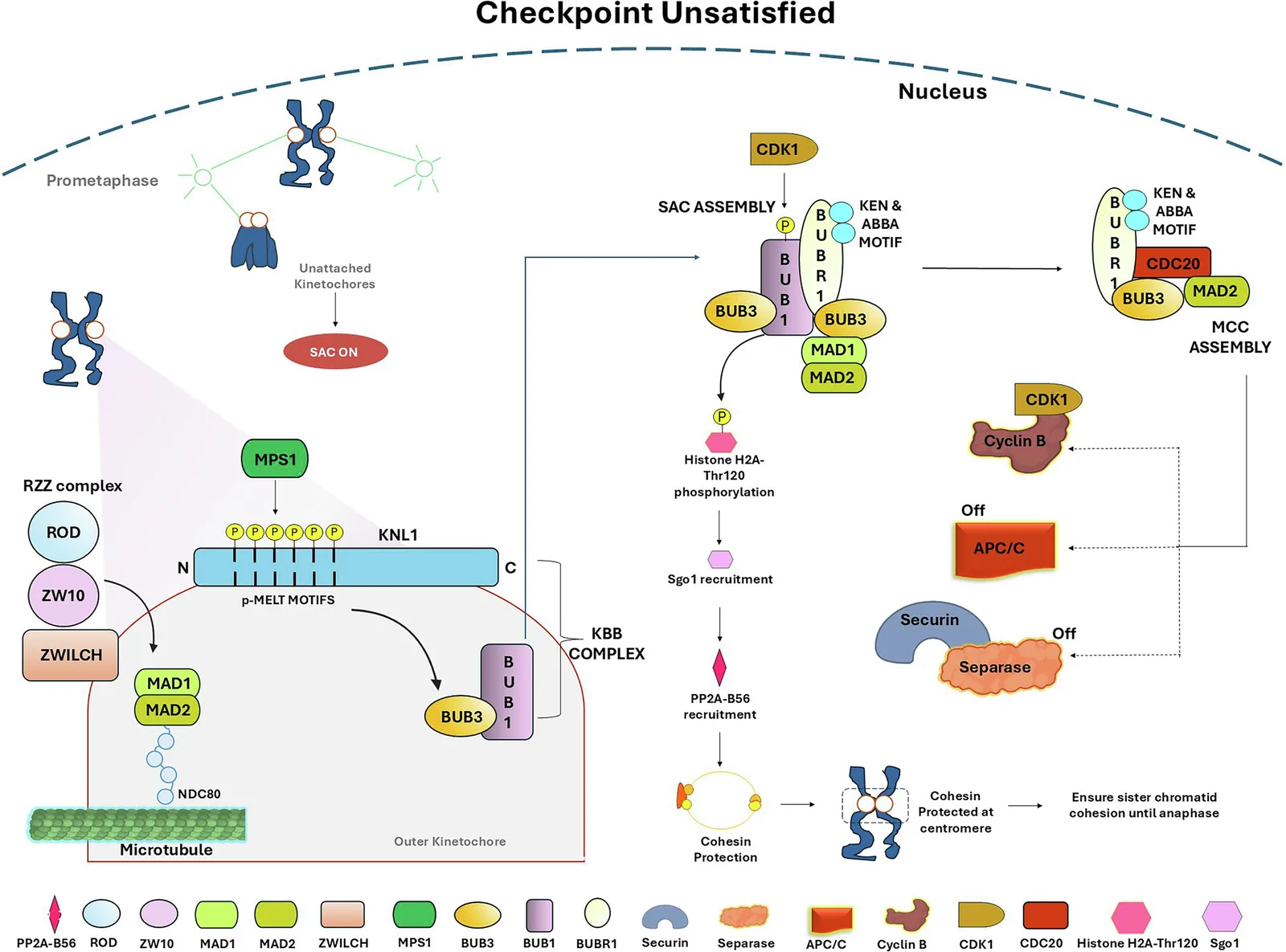
Mechanistic overview of SAC activation. The figure illustrates the molecular events that occur at unattached kinetochores during prometaphase to establish and maintain SAC Signaling. MPS1 phosphorylates the MELT motifs within KNL1, creating docking sites for the KNL1–BUB3–BUB1 (KBB) complex, while the RZZ (ROD–ZW10–ZWILCH) complex promotes recruitment of the MAD1–MAD2 complex to unattached kinetochores. CDK1-mediated phosphorylation further facilitates BUB1-dependent checkpoint assembly. Together, these events promote the formation of the MCC, comprising BUBR1, BUB3, MAD2, and CDC20, which sequesters CDC20 and inhibits APC/C activity, thereby preventing the degradation of cyclin B and securin and blocking premature anaphase onset. In parallel, BUB1 phosphorylates histone H2A at Thr120, leading to Shugoshin 1 (Sgo1) recruitment, PP2A-B56 localization, and protection of centromeric cohesin, thereby preserving sister chromatid cohesion until all chromosomes achieve proper kinetochore–microtubule attachment.

**Fig. 3:**
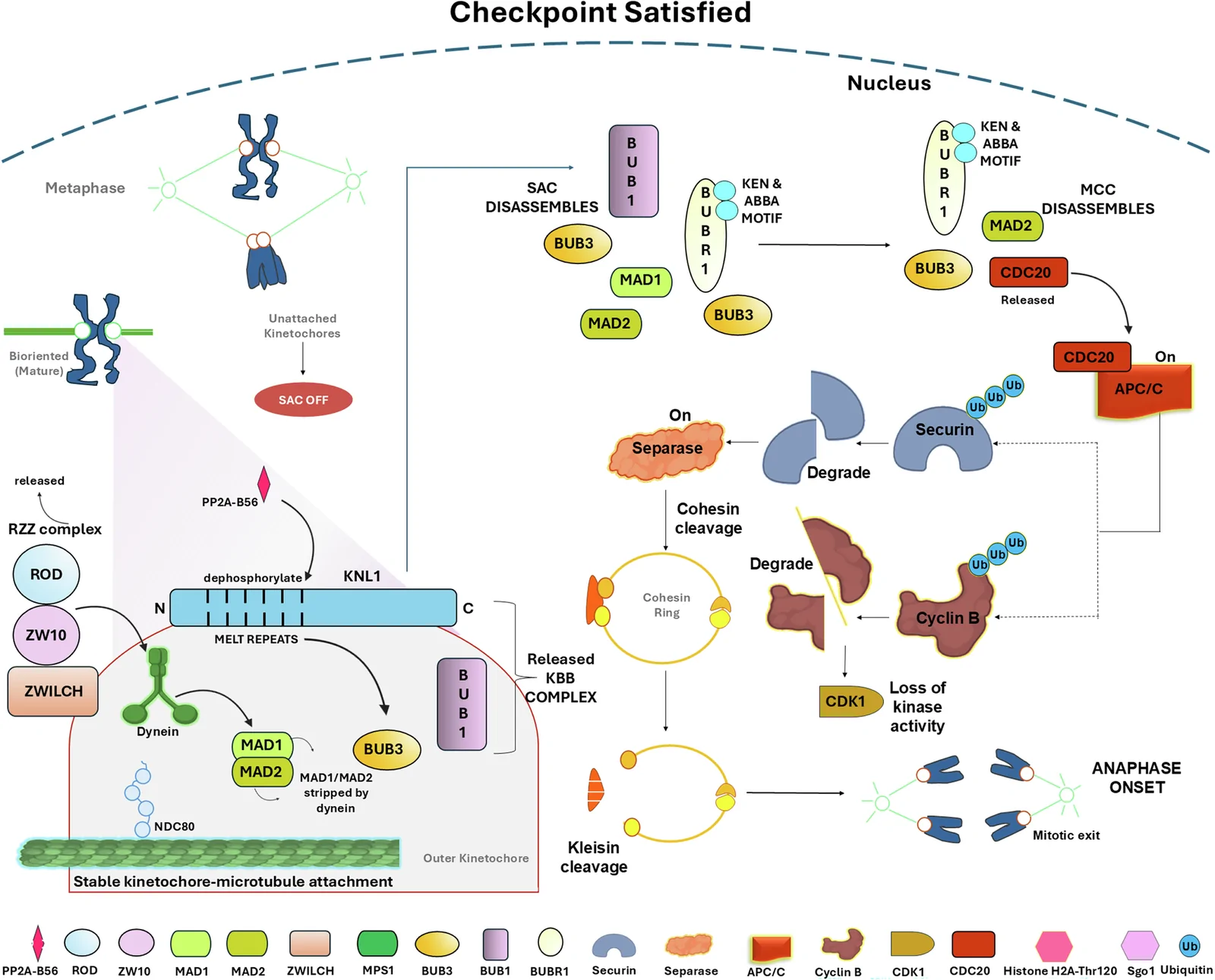
Mechanistic Overview of SAC silencing across mitotic progression. The figure illustrates the molecular events underlying SAC silencing following stable kinetochore–microtubule attachment during metaphase. Stable bi-orientation of sister chromatids displace MPS1 kinase from kinetochores, permitting PP2A-B56-mediated dephosphorylation of the KNL1 MELT motifs, dissociation of the KBB complex, and release of the RZZ complex from the kinetochore. Concurrently, dynein-mediated stripping removes the MAD1–MAD2 complex from kinetochores, resulting in SAC disassembly. These events promote the disassembly of the MCC, releasing CDC20 to activate the APC/C. Activated APC/C ubiquitinates securin and cyclin B, leading to their proteasomal degradation. Loss of securin activates separase, which cleaves the kleisin subunit of the cohesin complex, enabling sister chromatid separation and anaphase onset. Simultaneously, cyclin B degradation inactivates CDK1, facilitating mitotic exit and completion of chromosome segregation.

Accurate SAC signaling depends on the coordinated recruitment and dynamic regulation of BUB proteins at kinetochores, where they monitor microtubule attachment status and prevent premature anaphase onset. Fig. 2 illustrates the checkpoint-unsatisfied state, in which unattached kinetochores activate SAC signaling through sequential recruitment of BUB proteins, assembly of the MCC, and inhibition of APC/C–CDC20. In contrast, Fig. 3 depicts the checkpoint-satisfied state, where stable kinetochore–microtubule attachment triggers SAC silencing, MCC disassembly, APC/C activation, and mitotic progression into anaphase.

### BUB1-mediated checkpoint activation

Checkpoint activation is initiated through Monopolar Spindle 1 Kinase (MPS1)-dependent phosphorylation of MELT motifs within the kinetochore scaffold protein KNL1, generating docking sites for the BUB1–BUB3 complex via the GLEBS domain of BUB1^36^. Once recruited to kinetochores, BUB1 functions primarily as a scaffold that coordinates sequential assembly of SAC components, particularly the MAD1-MAD2 complex required for MCC formation and APC/C inhibition^4^. CDK1-mediated phosphorylation further enhances BUB1-dependent MAD1 recruitment and synergizes with MPS1 to promote robust SAC activation^36^. In addition to its scaffolding role, BUB1 contains APC/C Binding and Activation (ABBA) and KEN motifs that facilitate CDC20 interaction and checkpoint coordination^3^. Although its kinase activity is not absolutely essential for SAC initiation in mammalian cells, BUB1-mediated phosphorylation of H2A-T120, promotes Sgo1 recruitment, centromeric cohesion, and chromatin organization during mitosis^37^. BUB1 also contributes to spindle tension sensing through phosphorylation-dependent interactions with Aurora B kinase, thereby supporting correction of erroneous kinetochore-microtubule attachments and enhancing chromosome bi-orientation fidelity^38^. Through integration of upstream kinase signaling, kinetochore dynamics, and tension-responsive mechanisms, BUB1 functions as a central organizer of SAC activation and chromosome segregation accuracy.

### BUB1B as a regulator of checkpoint maintenance and chromosomal stability

BUB1B functions as a major inhibitor of APC/C-CDC20 activity and a crucial stabilizer of kinetochore-microtubule attachments. Similar to BUB1, BUB1B contains a GLEBS domain that mediates BUB3-dependent kinetochore localization, along with ABBA and KEN motifs required for MCC assembly and CDC20 binding^39^. However, unlike BUB1, BUB1B primarily exerts regulatory activity through its pseudokinase domain and KARD-mediated recruitment of PP2A–B56 phosphatase complexes^40^. Recruitment of PP2A-B56 counterbalances Aurora B kinase activity, thereby stabilizing kinetochore-microtubule attachments and preventing premature SAC silencing. Structural and functional studies further demonstrate that BUB1B acts as a scaffold for large checkpoint complexes that coordinate mitotic timing and chromosome alignment. Mouse models carrying hypomorphic BUB1B mutations exhibit mosaic variegated aneuploidy, CIN, and increased tumor susceptibility, underscoring its essential tumor-suppressive role in maintaining genomic integrity^41^. Consequently, BUB1B integrates checkpoint inhibition, phosphatase recruitment, and attachment stabilization to preserve mitotic fidelity and suppress CIN^42^.

### BUB3 as a scaffold for SAC stability

BUB3 functions as a conserved WD40-repeat scaffold protein that stabilizes kinetochore-associated checkpoint signaling^6^. Through phospho-dependent interactions with the GLEBS motifs of BUB1 and BUB1B, BUB3 facilitates their kinetochore recruitment and reinforces SAC assembly. In addition to promoting phospho-MELT recognition at KNL1, BUB3 supports outer kinetochore expansion through functional interactions with the RZZ complex^43,44^. Although catalytically inactive, BUB3 is essential for SAC integrity, as its depletion results in checkpoint failure, chromosome missegregation, and increased aneuploidy in dividing cells^45^. Beyond mitosis, emerging evidence implicates BUB3 in chromatin regulation, transcriptional repression, RNA processing, and epigenetic remodeling. Functional interactions with Rae1/Gle2 additionally link BUB3 to mRNA export, telomere maintenance, and aging-associated phenotypes in genetic models^46^. These findings position BUB3 not simply as a passive adapter, but as a dynamic organizer of checkpoint signaling and broader genome regulatory processes.

### Checkpoint silencing and mitotic exit

Following chromosome bi-orientation and stable spindle attachment, SAC signaling must be rapidly silenced to permit APC/C activation and mitotic exit. During prometaphase, checkpoint signaling is sustained through parallel recruitment pathways involving the KBB axis and the RZZ complex, both of which converge on MAD1–MAD2 recruitment^47^. As kinetochore attachment stabilizes, dynein-mediated stripping of checkpoint proteins and phosphatase-dependent dephosphorylation of MELT motifs by protein phosphatases PP1 and PP2A progressively dismantle SAC signaling^48,49^. BUB1B plays a pivotal role in this transition through KARD-dependent recruitment of PP2A-B56, which promotes kinetochore stabilization and checkpoint disassembly^40^. BUB1 also contributes to checkpoint silencing through regulation of phosphatase recruitment and centromeric cohesion maintenance^50^.

Importantly, BUB1B exists in two kinetochore-localized pools, including a BUB1- dependent population and a second pool that directly recognizes phosphorylated MELT motifs on KNL1. The latter contributes substantially to rapid SAC disassembly once chromosome bi-orientation is achieved^51^. Disruption of this silencing machinery promotes CIN, mitotic slippage, centrosome amplification, and p53-dependent stress responses that contribute to tumor evolution and therapeutic resistance^52^. Consistently, aberrant BUB1 and BUB1B expression correlates with poor clinical outcomes in multiple cancers, including breast cancer, thyroid cancer, hepatocellular carcinoma (HCC), lung adenocarcinoma (LUAD), and hematological malignancies^53–57^.

Emerging evidence further demonstrates that defective SAC silencing promotes chromothripsis, micronuclei formation, and immune evasion, linking mitotic dysregulation to tumor heterogeneity and inflammatory adaptation^58^. Beyond their canonical checkpoint functions, BUB proteins also participate in transcriptional regulation, DNA repair, RNA processing, and immune modulation. For example, BUB1B overexpression in LUAD drives metabolic reprogramming through interaction with the transcription factor (TF) zinc finger protein 143 (ZNF143), thereby enhancing glycolytic flux and tumor progression^59^. Functional enrichment analyses additionally support the conserved role of BUB proteins in chromosome segregation, sister chromatid cohesion, metaphase-to-anaphase transition, ubiquitin-mediated proteolysis, and spindle checkpoint regulation^60^, as shown in Supplementary Table 2. Together, these findings establish the BUB family as a multifunctional regulatory network that integrates mitotic surveillance with broader oncogenic and stress-response pathways.

## Beyond mitosis: non-canonical roles of BUB proteins

Although BUB proteins are classically recognized as core regulators of the SAC, accumulating evidence indicates that they also participate in diverse non-canonical processes beyond mitosis. These include transcriptional regulation, chromatin remodeling, DNA damage response (DDR), replication stress adaptation, and immune-associated signaling pathways that collectively influence tumor progression and cellular plasticity. Importantly, the functions of BUB proteins appear highly context-dependent, enabling them to act as both genome-protective and oncogenic regulators depending on cellular and tumor-specific conditions. The following sections summarize the emerging non-canonical roles of BUB proteins and their broader implications in cancer biology.

### Transcription factor and super-enhancer activity

Transcriptional deregulation is a defining feature of aggressive cancers and is frequently driven by oncogenic TFs, chromatin remodelers, and super-enhancer activation^61^. Super-enhancers consist of densely occupied enhancer clusters enriched for transcriptional coactivators and histone marks such as H3K27ac that sustain high-level expression of genes critical for tumor survival and proliferation^61^. Recent studies have identified BUB1B and BUB3 as components of super-enhancer-associated transcriptional programs in cancers, including triple-negative breast cancer (TNBC) and chronic myeloid leukemia (CML)^62,63^. These enhancer regions are transcriptionally governed by master regulators such as MYC, E2F1, and Forkhead Box M1 (FOXM1), generating feed-forward transcriptional circuits that sustain aberrant BUB expression and mitotic deregulation^64–66^. Mechanistically, BUB1B enhances E2F1 transcriptional activity, linking spindle checkpoint dysfunction with G1/S and G2/M transition deregulation^67^. Chromatin immunoprecipitation and sequencing (ChIP-seq) analyses further demonstrate enrichment of Bromodomain-Containing Protein 4 (BRD4) and H3K27ac at BUB1B-associated enhancer regions, supporting the existence of active oncogenic enhancer hubs^68,69^. BRD4-mediated enhancer activation reinforces MYC-FOXM1 signaling and amplifies proliferative transcriptional programs that promote CIN and tumor progression^66^.

**Fig. 4:**
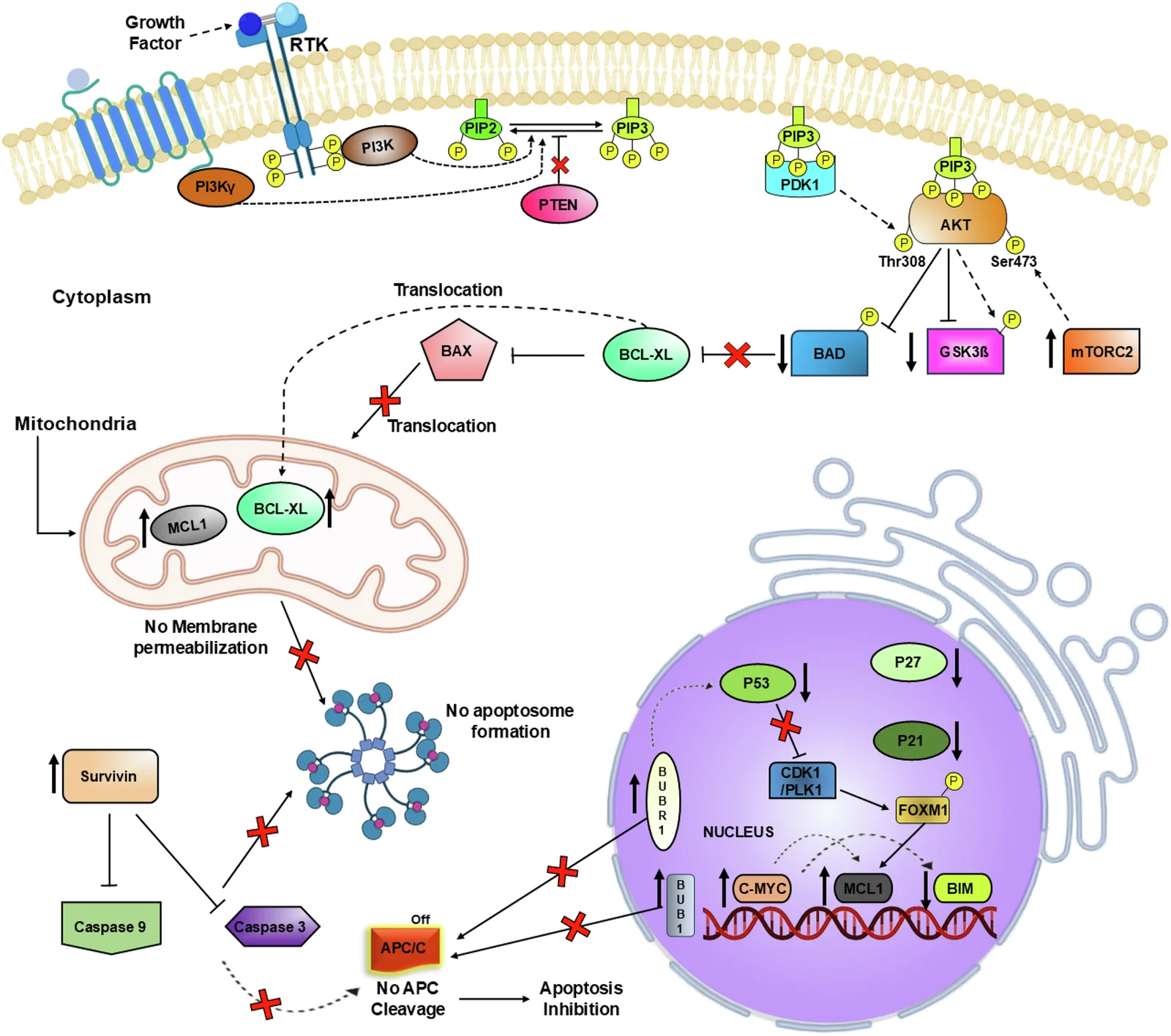
GPCR and RTK-mediated apoptosis evasion through PI3K–AKT signaling and BUB1/BUB1B activity in cancer cells. The figure illustrates how tumor cells evade intrinsic apoptosis through the integrated actions of G-protein coupled receptors (GPCRs), receptor tyrosine kinases (RTKs), PI3K–AKT signaling, and mitotic checkpoint proteins BUB1 and BUB1B. Upon ligand binding, both GPCRs and RTKs activate PI3K, which converts PIP2 to PIP3, a process negatively regulated by PTEN. PIP3 recruits and activates PDK1 and AKT, which is further phosphorylated by mTORC2. Activated AKT inhibits pro-apoptotic regulators (BAD, GSK3β) and upregulates anti-apoptotic proteins (BCL-XL, MCL1), blocking BAX-mediated mitochondrial membrane permeabilization. This prevents cytochrome c release, apoptosome formation, and activation of caspase-9 and caspase-3. Survivin contributes to caspase inhibition. In the nucleus, overexpressed BUB1B suppresses p53, p21, and p27, bypassing cell cycle arrest. BUB1, along with CDK1/PLK1, activates FOXM1, enhancing transcription of survival genes (C-MYC, MCL1) and downregulating pro-apoptotic BIM. BUB1B also inhibits APC/C activity, promoting mitotic persistence and chemoresistance. Together, GPCR/RTK-driven survival signaling and checkpoint dysregulation enable tumor cells to resist apoptotic cues and maintain proliferative advantage.

Beyond mitotic regulation, the MYC-E2F-FOXM1 axis contributes directly to therapeutic resistance. In tumors with MYC or FOXM1 amplification, elevated BUB1B expression becomes essential for tolerating genotoxic stress, supporting mitotic slippage, and sustaining tumor cell survival^70,71^. Genetic or pharmacological depletion of BUB1B in TNBC models impairs SAC fidelity, reduces cellular viability, and enhances sensitivity to DNA-damaging therapies^72^. Consistent with these findings, BUB1B-high tumors frequently exhibit resistance to taxanes and other genotoxic agents^73^. In addition, FOXM1 transcriptionally activates anti-apoptotic regulators including MCL1, BCL-XL, and Survivin, thereby reinforcing resistance to apoptosis during mitotic stress^74^. Collectively, the BUB1/BUB1B-CDK1/PLK1-FOXM1 signaling axis promotes cell-cycle progression while suppressing p53-, p21-, p27-, and caspase-mediated apoptotic responses^75,76^. Figure 4 summarizes this integrated mechanism through which checkpoint dysregulation and apoptotic resistance converge to support tumor progression and therapeutic evasion in BUB-overexpressing cancers.

Significantly, pharmacological inhibition of Bromodomain and Extra-Terminal Domain (BET) proteins using compounds such as JQ1 or OTX015 disrupts BRD4 occupancy at these enhancer sites and selectively suppresses BUB1B/BUB3 transcription, thereby sensitizing tumor cells to genotoxic stress and restoring checkpoint fidelity^69,77^. These studies position the BUB–super-enhancer axis as a critical regulatory interface linking mitotic dysfunction with oncogenic transcriptional amplification. A consolidated overview of BUB-mediated oncogenic signaling pathways across diverse tumor types is summarized in Supplementary Table 3.

### Epigenetic, post-transcriptional, and chromatin regulatory mechanisms

*BUB* gene expression in cancer is regulated through multiple interconnected epigenetic, post-transcriptional, and chromatin-associated mechanisms that collectively influence mitotic fidelity, CIN, and tumor progression. Promoter hypomethylation of BUB1 and BUB1B has been reported in hepatocellular and breast cancers, where it correlates with elevated expression, increased CIN, and poor prognosis^78,79^. Histone modifications, including enrichment of Histone H3 Lysine 4 Trimethylation (H3K4me3) at BUB1/BUB1B promoters, further contribute to transcriptional activation^80^. In addition, chromatin conformation studies, including Hi-C and ChIA-PET analyses, demonstrate that BUB1B loci are positioned within topologically associating domains (TADs) enriched for oncogenic enhancer interactions, thereby facilitating aberrant transcriptional activity^81^. At the epitranscriptomic level, N⁶-methyladenosine (m⁶A) modification enhances BUB1B mRNA stability under oxidative and genotoxic stress through YTH N6-Methyladenosine RNA Binding Protein 1 (YTHDF1)-dependent translational regulation^82^. Likewise, loss-of-function mutations in chromatin remodelers such as AT-Rich Interaction Domain 1A (ARID1A) and SWI/SNF Related, Matrix Associated, Actin Dependent Regulator of Chromatin, Subfamily A, Member 4 (SMARCA4) similarly alter enhancer accessibility and indirectly activate BUB1 and BUB3 transcription in ovarian and gastric cancers^83,84^.

At the post-transcriptional level, competing endogenous RNA (ceRNA) networks intricately fine-tune BUB expression. In HCC, Metastasis-Associated Lung Adenocarcinoma Transcript 1 (MALAT1) represses miR-193a-3p, increases BUB1 expression, and promotes tumor proliferation^85^. Additional RNA-binding proteins such as Insulin-Like Growth Factor 2 mRNA-Binding Protein 1 (IGF2BP1) stabilize BUB1B transcripts via m⁶A-dependent mechanisms, linking epitranscriptomic control to mitotic fidelity^86^. Under hypoxic conditions, circular RNAs (circRNAs) can regulate BUB1B expression by sponging inhibitory miRNAs, suggesting that ceRNA-mediated checkpoint regulation dynamically responds to tumor microenvironmental stress^87^.

Beyond transcriptional regulation, BUB1 also contributes directly to chromatin-associated mitotic signaling through coordination of Aurora kinase and PLK1 activity. Aurora B, a core component of the chromosomal passenger complex (CPC), regulates chromosome alignment, kinetochore–microtubule attachment, and cytokinesis during mitosis^88^. BUB1 phosphorylates H2A-T120, thereby creating a docking platform for Sgo1 and Aurora B recruitment at centromeres. Through this mechanism, BUB1 facilitates centromeric cohesion, tension sensing, and correction of faulty kinetochore attachments by the CPC^38^. In parallel, BUB1 cooperates with PLK1 in a mitotic feedback loop that integrates chromatin signaling with SAC fidelity to maintain accurate chromosome segregation^89^. Disruption of the BUB1–Aurora–PLK1 axis in cancer models results in CPC mislocalization, mitotic defects, and enhanced CIN, suggesting that this regulatory network may represent a therapeutic vulnerability in CIN-high tumors. Beyond centromeric chromatin regulation, emerging evidence suggests that BUB1 also contributes to telomere homeostasis. BUB1 has been implicated in maintaining telomere integrity and chromosome-end stability, further extending its role in preserving genome stability beyond canonical spindle assembly checkpoint signaling^90^. Although, the underlying molecular mechanisms remain to be fully elucidated.

Extending these findings, patient-derived organoid models integrated with single-cell epigenomic approaches, including the assay for transposase-accessible chromatin using sequencing (ATAC-seq), are emerging as valuable platforms for investigating chromatin remodeling, tumor heterogeneity, and therapeutic resistance in ARID1A/SMARCA4-deficient cancers^91^. These approaches may facilitate future studies aimed at defining how epigenetic alterations influence BUB1 and BUB3 regulation during tumor evolution, particularly in the context of ARID1A or SMARCA4 alterations^83^. Additionally, CRISPR Interference (CRISPRi)/CRISPR Activation (CRISPRa) screening approaches in organoid and Patient-Derived Xenograft (PDX) models are beginning to clarify these regulatory interactions, although in vivo functional validation remains limited^92^. Collectively, these findings position BUB proteins within a multilayered regulatory network linking chromatin remodeling, RNA stability, and mitotic adaptation during tumor evolution.

### Post-translational modifications

The localization, stability, and signaling activity of BUB proteins are tightly regulated by post-translational modifications (PTMs), including phosphorylation, ubiquitination, SUMOylation, and acetylation. These modifications function as dynamic molecular switches that coordinate checkpoint activation, kinetochore recruitment, and mitotic progression under physiological and stress-associated conditions^93^. Phosphorylation represents the most extensively characterized PTM within BUB signaling. BUB1 undergoes phosphorylation by kinases including MPS1 and Aurora B, thereby regulating kinetochore localization and SAC fidelity^94^. Similarly, phosphorylation of BUB1B at KEN-box and ABBA-associated regions modulates CDC20 interaction and APC/C inhibition, whereas PLK1-mediated phosphorylation enhances PP2A–B56 recruitment and checkpoint maintenance^39,95^. Additional PTMs contribute to spatial and temporal checkpoint regulation. Acetylation of BUB1B by the p300/CBP-associated factor (PCAF) stabilizes the protein during metaphase and prevents premature degradation^96^. Conversely, ubiquitination-mediated degradation of BUB proteins facilitates checkpoint silencing and mitotic exit^97^. Importantly, these PTMs operate within highly coordinated regulatory networks rather than as isolated events. These findings establish PTM-mediated regulation as a critical mechanism governing the dynamic signaling behavior of BUB proteins during mitosis and tumor adaptation.

### Crosstalk with tumor suppressors and oncogenic signaling

A pivotal aspect of BUB-driven tumor biology involves extensive crosstalk with tumor suppressor pathways and oncogenic signaling networks, particularly the p53-p21 axis. BUB1B is frequently overexpressed in chromosomally unstable tumors characterized by genotoxic stress and mitotic checkpoint defects^98^. Elevated BUB1B expression suppresses p21-mediated cell-cycle arrest and promotes continued proliferation despite genomic damage, especially in TP53-deficient settings^99^. This facilitates tolerance to aneuploidy and supports hyperproliferative phenotypes in multiple cancers, including endometrial, lung, breast, colorectal, ovarian, and HCCs^15,100–102^. BUB1 additionally forms regulatory feedback loops with oncogenic kinases such as PLK1 and AURKB, thereby reinforcing kinetochore signaling and chromatin organization^95,103^. In parallel, BUB1B cooperates with MYC to stabilize replication forks and suppress DNA damage accumulation during oncogenic stress, particularly in p53-deficient tumors^104^.

**Fig. 5:**
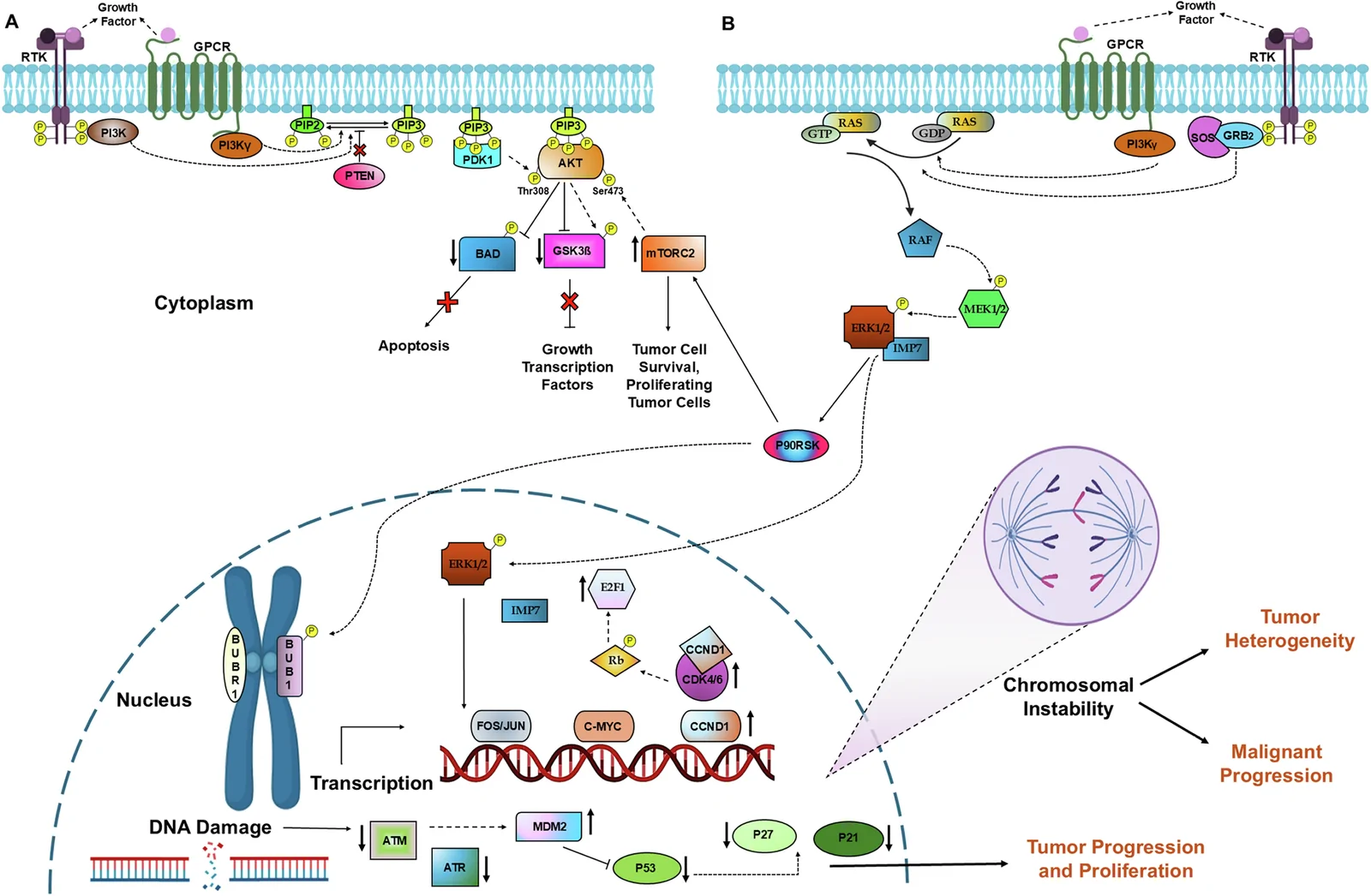
Oncogenic Signaling Pathways and the Role of BUB1/BUB1B Overexpression in Tumor Progression and Proliferation. The figure illustrates how overexpression of BUB1 and BUB1B (BUB1B) synergizes with oncogenic signaling initiated by both RTKs and GPCRs. In** A**, activation of the PI3K–AKT pathway via RTKs and GPCRs leads to phosphorylation of AKT, which inhibits pro-apoptotic proteins (e.g., BAD) and activates downstream targets like mTORC1 and GSK3β, promoting tumor cell survival and proliferation. GPCR signaling enhances PI3K recruitment and reinforces AKT activation, creating a growth-favorable environment. **B** Shows the RAS–RAF–MEK–ERK pathway, also activated by RTKs and GPCRs via intermediary effectors like Gβγ subunits and SOS/GRB2 complexes, leading to transcription of proliferative genes (FOS/JUN, C-MYC, E2F1) and cell cycle progression through CCND1–CDK4/6–Rb signaling. The ERK effector p90RSK further augments this by phosphorylating nuclear targets, including BUB1, thereby strengthening oncogenic transcriptional outputs. Within the nucleus, overexpressed BUB1/BUB1B suppress the tumor suppressor axis (p53, p21, p27), override DNA damage responses, and promote CIN by weakening spindle checkpoint fidelity. CIN accelerates tumor heterogeneity and malignant progression. GPCRs thus function as key amplifiers of mitogenic signals converging on BUB1/BUB1B-driven oncogenic circuits.

Beyond their canonical mitotic roles, BUB1 and BUB1B also modulate non-canonical pathways such as Wingless-related integration site/beta-catenin (Wnt/β-catenin), phosphoinositide 3-kinase (PI3K)/protein kinase B (AKT), and mitogen-activated protein kinase (MAPK)/extracellular signal-regulated kinase (ERK) signaling^105–108^. Aberrant BUB1B activity promotes β-catenin nuclear localization and downstream transcriptional activation, driving EMT, stemness, and metastasis in cancers such as bladder cancer and chronic lymphoid leukemia^109,110^. Furthermore, as illustrated in Fig. 5, BUB-driven CIN may further amplify PI3K/AKT and MAPK/ERK signaling, thereby promoting proliferation, immune evasion, and survival under therapeutic stress. Collectively, these findings position BUB proteins as integrators of mitotic surveillance and oncogenic signaling networks that support tumor progression and adaptive stress responses.

### Immunomodulation by BUB proteins

Recent studies have increasingly linked BUB dysregulation to remodeling of the tumor immune microenvironment (TIME) and immune checkpoint signaling^111,112^. Integrative multi-omics analyses have shown that aberrant expression of BUB1B correlates strongly with upregulation of immune checkpoints (PD-L1, CTLA-4, and LAG3) across multiple tumor types^53,113^. Similarly, pan-cancer transcriptomic analyses have associated elevated BUB3 expression with altered immune infiltration patterns in breast and lung cancers. However, because BUB3 functions primarily as a scaffold protein within the SAC, whether it directly regulates these immune alterations or simply reflects broader mitotic dysregulation remains unresolved^10,22^. Mechanistically, BUB1B-driven CIN promotes micronuclei formation and cytosolic DNA release, thereby activating the cyclic GMP-AMP synthase–stimulator of interferon genes (cGAS–STING) pathway and inducing type I interferon signaling^111,114^. However, persistent CIN may paradoxically sustain chronic inflammatory signaling, that promotes macrophage polarization, T-cell dysfunction, and immune exhaustion.

From a translational perspective, these observations suggest that BUB expression signatures may predict responsiveness to immune checkpoint blockade, particularly in tumors characterized by high CIN and immune exhaustion. Combined targeting of mitotic checkpoint signaling and immunotherapy may therefore represent a promising strategy for treatment-resistant cancers. Additionally, beyond their roles in immune modulation, BUB proteins continue to contribute to DNA damage sensing and genome maintenance under genotoxic stress^115^. Collectively, these findings position the BUB family at the intersection of mitotic fidelity, inflammatory signaling, genome instability, and tumor immune adaptation. However, several observations discussed above, including the associations of BUB1/BUB1B expression with immune checkpoint upregulation and of BUB3 expression with altered immune infiltration, are derived primarily from transcriptomic and bioinformatic analyses. Consequently, additional in vivo and functional studies are required to establish direct causal roles for BUB proteins in immune regulation and tumor progression.

## BUBs-associated signaling pathways

**Fig. 6:**
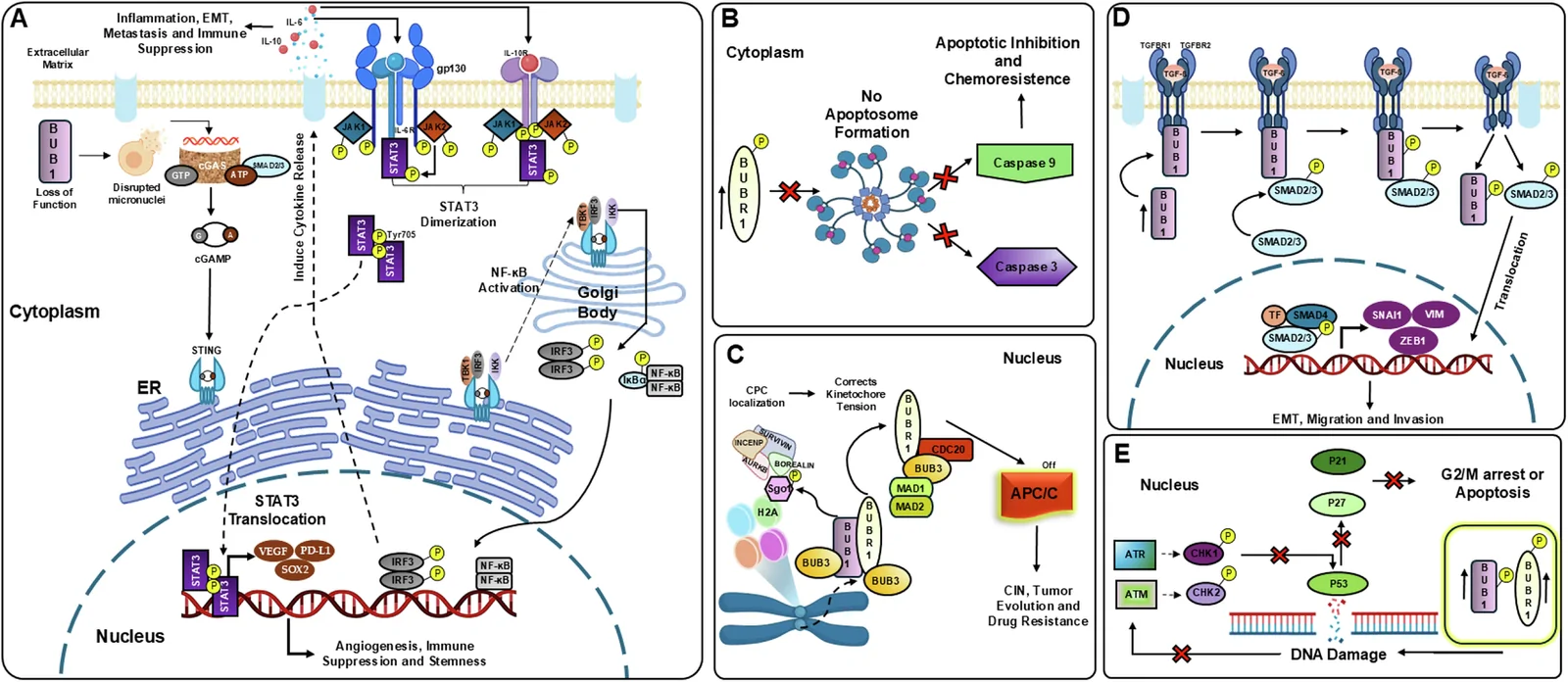
BUB- driven signaling pathways in cancer. The figure illustrates the multifaceted oncogenic roles of BUB1 and BUB1B in promoting tumor progression, immune evasion, drug resistance, and inhibition of apoptosis. In **A** BUB1 loss disrupts microtubule integrity and activates the cGAS–STING pathway, triggering STAT3 and NF-κB signaling that promote inflammation, immune suppression, epithelial–mesenchymal transition (EMT), and metastasis. In **B** Phosphorylated BUB1B inhibits apoptosome formation, suppressing caspase-9 and caspase-3 activation and conferring chemoresistance. In** C** BUB1–BUB3 complexes correct kinetochore tension but can also promote persistent mitotic arrest by maintaining MCC and inhibiting APC/C, leading to CIN, tumor evolution, and drug resistance. In **D** BUB1 facilitates TGF-β–SMAD2/3 nuclear translocation and activation of EMT transcription factors (SNAI1, ZEB1, VIM), driving migration and invasion. In **E** Overexpressed BUB1B suppresses p53, p21, and p27 despite DNA damage, thereby bypassing the p53-mediated G2/M checkpoint. When DNA damage persists, suppression of p53 signaling also impairs apoptosis, promoting survival under genotoxic stress. Collectively, these mechanisms enable cancer cells to evade cell-cycle checkpoints and apoptosis, thereby promoting tumor progression, therapeutic resistance, and aggressive behavior.

Advances in molecular oncology and systems biology have expanded the understanding of BUB proteins beyond their canonical mitotic functions. Emerging evidence further indicates that BUB signaling intersects with pathways governing DDR, oncogenic transcriptional programs, immune checkpoint regulation, and CIN-associated inflammatory signaling^116,117^. This section summarizes the major signaling pathways associated with BUB family proteins and highlights their mechanistic, pathological, and translational relevance in cancer biology. Figure 6 provides an integrated schematic of these signaling interactions, emphasizing how BUB1, BUB1B, and BUB3 interface with major pathways, including the Transforming Growth Factor Beta/SMAD signaling Pathway (TGF-β/SMAD), NF-κB, DDR, apoptotic regulation, and transcriptional rewiring via FOXM1 and MYC.

### TGF-β/SMAD pathway

The TGF-β pathway is a key regulator of EMT, immune modulation, and tumor progression^118,119^. Canonically, TGF-β signaling involves ligand-induced phosphorylation of SMAD2 and SMAD3 by TGFBR1, promoting their nuclear translocation and transcriptional activation of target genes^120^. Recent evidence, however, has identified BUB1 as an unexpected regulator of this pathway. A landmark study by Nyati et al. demonstrated that BUB1 is recruited to the TGFBR1 complex following ligand stimulation, where its kinase activity facilitates SMAD2/3 activation and downstream transcriptional signaling. Notably, BUB1-deficient or kinase-dead cells exhibit impaired SMAD signaling despite intact upstream TGF-β receptors, highlighting a non-canonical role for BUB1 in TGF-β pathway regulation^121^.

Mechanistically, BUB1 appears to function in coordination with ALK5 to modulate SMAD dynamics and transcriptional responses under oncogenic conditions^122^. Elevated BUB1 expression has been reported in TNBC, NSCLC, and HCC, where hyperactive TGF-β signaling is associated with EMT, metastasis, and chemoresistance^122–124^. In these contexts, BUB1 promotes the expression of EMT-associated genes, including SNAI1, ZEB1, and VIM, thereby facilitating invasive tumor phenotypes^108,125^. These findings position BUB1 as a context-dependent regulator of TGF-β/SMAD signaling and further support the emerging concept that mitotic checkpoint proteins can acquire non-mitotic signaling functions that contribute to tumor progression.

### Inflammation and NF-κB signaling

Emerging evidence suggests that BUB1 contributes to tumor-promoting inflammation through activation of NF-κB-associated signaling pathways. In cancer cells, BUB1 overexpression has been linked to activation of tumor necrosis factor receptor-associated factor 6 (TRAF6), which subsequently promotes NF-κB signaling and transcription of genes involved in inflammation, cell survival, proliferation, and metastasis^126^. Persistent NF-κB activation is a well-established driver of chronic inflammation within the TME, thereby supporting tumor progression and immune evasion^127^. In addition, chromosome missegregation induced by defective BUB1 signaling promotes micronuclei formation, and rupture of these micronuclei releases chromatin fragments into the cytosol, activating the cGAS-STING innate immune sensing pathway^128–130^. Subsequent activation of NF-κB stimulates production of pro-inflammatory cytokines, including IL-6, IL-8, and TNF-α, thereby linking mitotic stress to inflammatory signaling and tumor-associated immune remodeling. In multiple cancers, including colorectal, lung, and gastric tumors, elevated BUB1 expression correlates with increased expression of NF-κB target genes and inflammatory signatures^126,131^.

Functionally, chronic activation of the cGAS–STING–NF-κB axis contributes to senescence-associated secretory phenotype (SASP) signaling, extracellular matrix remodeling, EMT, and immunosuppressive TME formation, all of which promote tumor invasiveness and metastatic progression^132–134^. These mechanisms appear particularly relevant in TP53-deficient tumors, where defective cell-cycle control enhances dependence on inflammatory signaling pathways for tumor survival^130^. Importantly, preclinical studies demonstrate that STING inhibition can attenuate NF-κB-driven inflammation and suppress SASP-associated tumor progression, highlighting the translational significance of the BUB1-CIN-inflammation axis^135,136^. Nevertheless, although the association between BUB1 dysregulation, micronuclei formation, and inflammatory signaling is compelling, additional mechanistic studies are needed to establish direct causality and therapeutic relevance.

### DNA Damage Response (DDR)

In addition to its canonical role in SAC signaling, BUB1 has emerged as an important regulator of the DDR, particularly under conditions of genotoxic stress^137^. Following DNA double-strand breaks (DSBs), BUB1 is recruited to damaged chromatin, where it contributes to early DDR signaling through regulation of γH2AX formation and H2A-T120 phosphorylation. BUB1 functions downstream of the ATM and ATR kinases and facilitates recruitment of Sgo1 while supporting chromatin remodeling and G2/M checkpoint enforcement under genotoxic conditions^138^. ATM/ATR-mediated signaling also promotes activation of downstream checkpoint effectors, including CHK2 and p53, thereby coordinating cell-cycle arrest with DNA repair responses^139^. These findings highlight a functional link between mitotic checkpoint regulation and genome maintenance in rapidly proliferating tumor cells.

In parallel, BUB1B contributes to the anti-apoptotic arm of the DDR by suppressing caspase-3 and caspase-9 activation during prolonged mitotic arrest^140^. Elevated BUB1B expression has therefore been associated with resistance to DNA-damaging agents such as cisplatin and doxorubicin in multiple cancers. Functional studies in hypomorphic BUB1 mouse models further demonstrate that partial BUB1 depletion sensitizes cells to genotoxic stress, resulting in increased γH2AX accumulation and enhanced apoptosis following DNA damage^7^. Moreover, interactions between BUB1 and p53 appear highly context-dependent; while BUB1 may support p53-mediated checkpoint responses, BUB1 loss in p53-deficient settings can exacerbate genomic instability and accelerate tumor progression, suggesting a potential synthetic lethal relationship between these pathways^141^.

Collectively, these findings position BUB1 as a key integrator of mitotic checkpoint signaling and DNA damage surveillance. Targeting BUB-associated DDR pathways may therefore represent a promising strategy for chemosensitization and synthetic lethality in tumors characterized by impaired p53 signaling or elevated replication stress.

## Therapeutic landscape: targeting the BUB family

The BUB family has emerged as a promising therapeutic target in cancer because of its critical role in SAC regulation, chromosomal stability, and tumor progression. Dysregulated BUB signaling is strongly associated with CIN, therapy resistance, immune remodeling, and poor prognosis across multiple cancers. Consequently, inhibitors targeting BUB1, BUB1B, and related SAC pathways are being explored as potential precision oncology strategies. Recent advances in structure-based drug design, allosteric inhibition, and high-throughput screening have enabled the development of increasingly selective BUB-targeted compounds. Although no BUB-directed therapies have yet entered clinical trials, preclinical studies indicate considerable potential for both monotherapy and combinatorial therapeutic approaches. The following sections summarize the major pharmacological inhibitors targeting BUB family proteins, while Supplementary Table 4 provides an overview of these compounds and Supplementary Table 5 summarizes their clinical and translational significance across tumor types.

### Inhibitors targeting BUB1

#### BAY 1816032

BAY 1816032 is a potent and selective ATP-competitive inhibitor of BUB1 developed by Bayer^142^. It suppresses BUB1 kinase activity by blocking H2A–T120 phosphorylation, thereby impairing CPC recruitment during mitosis^143^. Preclinical studies have demonstrated strong antitumor activity, particularly in chromosomally unstable cancers such as triple-negative breast and ovarian cancers, where it exhibits synergistic effects with paclitaxel and PARP inhibitors^143–145^. BAY 1816032 also demonstrates high kinase selectivity, favorable pharmacokinetic properties, and a promising preclinical safety profile, supporting its potential as a therapeutic strategy targeting mitotic checkpoint vulnerabilities in cancer^143^.

#### BAY–320

BAY–320 is an ATP-competitive inhibitor of BUB1 that disrupts SAC signaling by inhibiting BUB1-dependent phosphorylation of H2A-T120, a modification required for Sgo and CPC recruitment at centromeres^146,147^. Inhibition of this pathway leads to defective kinetochore signaling, displacement of CPC components, and impaired mitotic fidelity. Although BAY-320 demonstrated high selectivity and potent activity in cell-based studies, its poor pharmacokinetic profile limited further in vivo and clinical development^146^. Nevertheless, it has served as an important tool compound for elucidating BUB1-mediated mitotic signaling and has shown synergistic anti-proliferative effects with low-dose paclitaxel, particularly in chromosomally unstable and aneuploid cancer cells^147^.

#### BAY–524

BAY–524 is a structurally optimized analog of BAY-320 developed to improve kinase selectivity and cellular potency. It disrupts SAC signaling by inhibiting BUB1-mediated H2A-T120 phosphorylation and impairing CPC localization at centromeres^147^. Compared with BAY-320, BAY-524 demonstrates enhanced biochemical and cell-based efficacy and has been widely used in preclinical synthetic lethality studies, particularly in cancers with defective DNA repair or elevated CIN^148^. Additionally, combination treatment with paclitaxel promotes chromosome missegregation and mitotic failure, highlighting its potential utility for targeting CIN-dependent tumors^147^.

#### 2OH-BNPP1

2OH-BNPP1 (2-hydroxy-benzylidene-nitro-phenyl-prop-2-en-1-one) is a chalcone-derived small molecule reported to inhibit BUB1 kinase activity. Functionally, it disrupts BUB1-dependent centromeric phosphorylation events, thereby impairing SAC signaling and promoting mitotic errors and CIN^146^. However, 2OH-BNPP1 also exhibits inhibitory activity against other kinases, including PDGFRB and VEGFR2, indicating limited selectivity and restricting its translational potential as a BUB-targeted therapeutic agent. Consequently, it is primarily regarded as a multi-kinase chemical probe for investigating BUB1-associated mitotic signaling pathways rather than a clinically relevant inhibitor^143^.

### Indirect modulators of BUB1B-associated checkpoint signaling

Although no selective direct BUB1B inhibitors have been reported to date, several compounds indirectly disrupt BUB1B-mediated spindle checkpoint signaling by targeting upstream mitotic regulators.

#### ZM447439

ZM447439 is a well-characterized Aurora kinase inhibitor that indirectly disrupts BUB1B-mediated SAC activity by impairing kinetochore–microtubule attachment and tension sensing^149,150^. Through inhibition of Aurora A and B kinases, the compound induces mitotic slippage and defective chromosome segregation, processes closely linked to BUB1B checkpoint function. ZM447439 has demonstrated significant antitumor activity in multiple preclinical models of chromosomally unstable cancers and remains an important tool compound for studying SAC regulation.

#### TAK-960

TAK-960 is a potent PLK1 inhibitor that indirectly impairs BUB1B function through disruption of mitotic spindle assembly and kinetochore organization^151–153^. Inhibition of PLK1 interferes with proper checkpoint signaling, leading to mitotic defects, checkpoint override, and tumor cell apoptosis. TAK-960 has shown broad antiproliferative activity across several solid tumor models and advanced into Phase I clinical evaluation before clinical development was discontinued^154^. Nevertheless, it remains of translational interest for targeting mitotic vulnerabilities in cancer.

### Reversine

Reversine is a synthetic purine analog with pan-Aurora kinase and Mps1 inhibitory activity that induces SAC failure and CIN^155,156^. By disrupting kinetochore–microtubule attachment and checkpoint signaling, Reversine indirectly compromises BUB1B-associated mitotic control, resulting in aneuploidy and apoptotic cell death. Although still limited to preclinical investigation, Reversine is widely used as an experimental agent for studying checkpoint fidelity and CIN under mitotic stress^157^.

### Indirect modulators of BUB3-associated checkpoint signaling

To date, no direct pharmacological inhibitors of BUB3 have been identified. However, disruption of proteins required for BUB3-dependent checkpoint function indirectly compromises SAC signaling.

#### GSK923295

GSK923295 is a high-affinity inhibitor of the kinetochore motor protein CENP-E that indirectly destabilizes BUB3-associated checkpoint complexes^158^. By disrupting chromosome alignment and microtubule dynamics, the compound compromises SAC integrity and interferes with the stabilization of kinetochore checkpoint signaling mediated by BUB3-containing complexes. Although clinical development was discontinued following Phase I evaluation, GSK923295 demonstrated measurable antitumor activity and provided important insights into targeting mitotic checkpoint vulnerabilities in cancer^159^.

### Synthetic lethality and combinatorial therapeutic strategies associated with BUBs

Synthetic lethality has emerged as a promising precision oncology strategy in which simultaneous disruption of complementary survival pathways selectively induces tumor cell death while sparing normal cells^160^. This approach is particularly relevant in cancers with CIN or defects in DDR, where SAC regulators such as BUB1, BUB1B, and BUB3 become essential for maintaining mitotic fidelity and tumor survival^6,22,161^. Through regulation of chromosome alignment, kinetochore–microtubule attachment, and checkpoint activation, BUB proteins help cancer cells tolerate elevated genomic instability. Consequently, therapeutic targeting of BUB-associated pathways has gained increasing attention as a strategy to selectively exploit mitotic vulnerabilities in genetically unstable tumors. Among these approaches, combined inhibition of BUB1 and PARP has shown considerable therapeutic promise. BUB1 regulates H2A-T120 phosphorylation to facilitate centromeric recruitment of Sgo1 and Aurora B during mitosis^162^. Pharmacological inhibition of BUB1 disrupts these processes, leading to defective chromosome segregation and impaired checkpoint fidelity. In parallel, PARP inhibitors promote accumulation of unrepaired DNA damage in homologous recombination (HR)-deficient tumors by preventing repair of single-strand DNA breaks^163^. Concurrent inhibition of BUB1 and PARP drives mitotic progression despite unresolved DNA damage, thereby enhancing CIN, micronuclei formation, and apoptotic cell death, particularly in BRCA-deficient and TNBC models^143–145^.

Additional combinatorial strategies involve co-targeting SAC-associated kinases such as PLK1 and Aurora kinases, which function cooperatively with BUB proteins to maintain chromosome segregation fidelity. Mechanistically, BUB1 recruits PLK1 to kinetochores to facilitate kinetochore maturation, while Aurora B kinase regulates correction of erroneous microtubule attachments during mitosis^162^. Simultaneous inhibition of these pathways using agents such as BI 2536, volasertib, or barasertib has demonstrated synergistic antitumor effects in CIN-high tumors by amplifying mitotic stress, disrupting checkpoint signaling, and promoting mitotic catastrophe^164–166^. These findings suggest that multi-node disruption of SAC machinery may represent an effective strategy for overcoming compensatory signaling and therapeutic resistance in highly unstable cancers. Beyond DDR-deficient tumors, BUB-centered synthetic lethality may also extend to MYC-amplified, KRAS-mutant, and CDK12-deficient cancers, which exhibit a strong dependency on accurate mitotic progression^167–169^. Moreover, emerging translational strategies integrating multi-omics profiling, computational network modeling, and patient-derived organoid systems may facilitate identification of predictive biomarkers such as CIN signatures and BUB expression patterns for patient stratification^170^. Collectively, these studies highlight the therapeutic potential of exploiting BUB-associated synthetic lethal interactions as a next-generation strategy in precision oncology.

## Translational relevance and future directions

The BUB family has emerged as an important regulator of cancer biology with growing clinical and translational significance beyond its canonical role in mitotic checkpoint control^171^. Functionally, tumors with high BUB expression often exhibit poor clinical outcomes, resistance to conventional chemotherapeutic agents such as taxanes, while displaying increased sensitivity to DNA damage repair-targeted therapies, including PARP inhibitors, particularly in TP53- or BRCA1/2-deficient backgrounds^172,173^. Thus, these findings support the emerging role of BUB proteins as both prognostic and predictive biomarkers in precision oncology.

Beyond their prognostic significance, BUB proteins are increasingly recognized as active participants in oncogenic signaling networks. BUB1 cooperates with PLK1 to promote spindle checkpoint signaling through CDC20 phosphorylation and has also been reported to exhibit coordinated expression with AURKA and AURKB in ovarian serous carcinoma, whereas BUB1B activates Wnt/β-catenin signaling to promote tumor proliferation and metastasis^95,174–176^. ceRNA network analyses further identify regulatory axes such as LINC01184–miR-22–BUB1B and MALAT1–miR-495-3p–BUB1, highlighting additional epigenetic and post-transcriptional mechanisms contributing to BUB dysregulation in cancer^177–179^. Functional studies using CRISPR-Cas9, knockout systems, and genome-wide dependency screens further demonstrate that BUB1 and BUB1B function as context-specific essential genes in multiple cancers, including TNBC and malignant mesothelioma^57,180^. While BUB1 loss induces kinetochore defects, aneuploidy, and mitotic slippage, depletion of BUB1B produces more severe phenotypes associated with checkpoint collapse and enhanced tumor susceptibility^102,181–183^. Although catalytically inactive, BUB3 remains indispensable for mitotic checkpoint integrity through stabilization of the MCC^184^.

Therapeutically, targeting BUB-associated signaling pathways has gained considerable interest as a strategy for exploiting mitotic vulnerabilities in CIN-high tumors. Small-molecule inhibitors targeting BUB1, BUB1B-associated pathways, Aurora kinases, and PLK1 have demonstrated promising preclinical antitumor activity through induction of mitotic catastrophe, checkpoint failure, and apoptotic cell death^151,164,165^. Combination-based strategies involving PARP inhibitors, microtubule-targeting agents, and immune checkpoint blockade have further highlighted the potential of BUB-centered synthetic lethality approaches, particularly in genomically unstable cancers^185–190^. In addition, emerging therapeutic platforms such as PROTAC-mediated degradation, CRISPR-dCas-based epigenetic modulation, nanoparticle delivery systems, and exosome-mediated targeting may improve specificity while reducing systemic toxicity associated with mitotic checkpoint inhibition^191,192^. However, clinical translation remains challenging because BUB proteins are also essential for mitosis in normal proliferative tissues, including bone marrow and intestinal epithelium, resulting in narrow therapeutic windows and potential on-target toxicities^193,194^.

Recent technological advances are expected to accelerate the clinical translation of BUB-directed therapies. Single-cell sequencing, spatial transcriptomics, super-resolution imaging, and integrative multi-omics analyses are providing deeper insights into BUB-dependent signaling dynamics, tumor heterogeneity, and immune interactions within the TME^195,196^. Concurrently, artificial intelligence (AI)- and machine learning (ML)-based approaches are improving biomarker discovery, predictive modeling, and structure-guided drug development, including identification of novel binding pockets for next-generation therapeutics^197–199^. More physiologically relevant preclinical systems, including patient-derived organoids and xenograft models, may further facilitate evaluation of BUB-targeted therapies in CIN-driven cancers while enabling patient stratification based on molecular vulnerabilities such as TP53 loss, MYC amplification, KRAS mutation, or CDK12 deficiency. Circulating RNA signatures and immune-associated biomarkers linked to BUB signaling may additionally provide non-invasive tools for monitoring disease progression and therapeutic response.

### Reporting summary

Further information on research design is available in the Nature Portfolio Reporting Summary linked to this article.

## Supplementary information

Transparent Peer Review file

SUPPLEMENTAL MATERIAL

Reporting Summary

## Data Availability

No datasets were generated or analysed during the current study. The abbreviation list is provided as Supplementary Table 6.
