# Supplementary material for: BUB signaling in cancer and molecular insights into targeted therapies
Source: Commun Biol. 2026 Oct 16;9:1335. doi: 10.1038/s42003-026-10976-5 (PMC13675223; doi:10.1038/s42003-026-10976-5)
Supplement: Supplementary file 1 [file 42003_2026_10976_MOESM1_ESM.pdf]

nature portfolio

Peer Review File

BUB signaling in cancer and molecular insights into targeted therapies

Corresponding Author: Dr Rajeev Nema

This file contains all reviewer reports in order by version, followed by all author rebuttals in order by version.

Version 1:

Reviewer comments:

Reviewer #1

(Remarks to the Author)
This manuscript presents an interesting and extensive revision of the involvement of human BUB proteins, key components of the spindle assembly checkpoint (SAC) signalling network, in oncogenesis and their potential as novel therapeutic targets for the treatment of cancer. A strength of the manuscript is the analysis of the cross-talk mechanisms between BUBs and other signalling pathways such as NF-κB and TGF-β/SMAD. The topic is important and timely and could be a useful reference for those interested in the study of molecular aspects of BUB functions beyond the SAC, the association of these multidomain proteins with cancer as well as their potential to develop novel cancer therapeutics. Overall, the manuscript is organised in logical progression, with an acceptable narrative flow. However, there are some important inaccuracies, figures that need improvement, repetition of themes and grammar and typos and general presentation issues that must be addressed before the manuscript can be considered for its publication in Communications Biology. There are summarised as follows:

BubR1 and BUB1B are used throughout the manuscript to refer to the same protein. For the sake of clarity, I recommend mentioning at the beginning of the manuscript that an alternative nomenclature exist (either BubR1 or BUB1B) and use only one of such names afterwards.

Section 2.0 Evolution and Structural Organization. Because a proper evolutionary analysis of BUBs requires a thorough comparison of BUBs orthologues and paralogues across species, I feel this topic distracts the attention from the main aim of this work. Namely, an in depth analysis of the impact of human BUB proteins in oncogenic processes and their potential use in cancer therapeutics. The fact Figure 1 shows a wrong description of domains in Bub3, does not help to mitigate my concerns about the limited value of this section. Bub3 is a globular protein mostly organised as a 7-blade beta propeller rather than in two large regions of low structural complexity as the authors claim in this figure (admittedly, in the main text the authors refer to the WD40 fold when discussing Bub3 structure). There is nothing in Figure 1 that explains gene duplication events in the Bubs, really.

Figure 2 needs improvement. The caption lacks a proper description of what this figure intents to convey (for example, Sgo1 is shown in the two cartoons but it was not even mentioned in the figure legend). The MELT motifs of KNL1 appear near the C-terminal end when they should be shown in KNL1 N-terminal end.

Figure 3 shows essentially the same information shown in Figure 2: the series of events that drive a SAC response. The same can be said of Figure 6 Panel C.

Inhibitors targeting BubR1 and Bub3. This is misleading as none of the molecules described actually target BubR1 nor Bub3 directly. For instance, TAK-960 was described as an inhibitor of BubR1. However, this compound is a PLK1 inhibitor. Similarly, ZM447439 is an Aurora kinase inhibitor. Sections 6.2 and 6.3 must be rewritten completely or removed.

For clarity, the list of abbreviations must be presented in alphabetical order.
APC and APC/C are mentioned twice. The mention of the use of the APC acronym in a different context (and therefore meaning) is not relevant to this work and should be removed.
In the list of abbreviations each protein should be listed separately. For instance, interleukine 6 and interleukine 8 rather than IK6 and 8; Caspase-3 and Caspase-9.

Some acronyms are incorrect. For example Survivin Baculoviral IAP Repeat Containing 5 (BIRC5), AKT protein kinase B and p65.

Some statement lack scientific rigour; A few examples are:
"(BUBR1) C-terminal kinase domain primarily functions as a pseudokinase. More description of what this mean seems appropriate.
"Tumor protein p53" (p53 is a tumour supressor protein)
4.2: "Extending these findings, resistant organoid models..." Resistant to what exactly? This should be spelled out.
4.5: "Although BUB3 lacks catalytic activity, its elevated expression in immunologically "cold" tumors such as breast and lung cancers suggests potential involvement in immune exclusion and transcriptional adaptation" how exactly Bub3 may be involved in these processes? This needs clarification.
Also "However, many current studies remain correlative" please mention specific examples here.
Sections 6.2 and 6.3 Again, the molecules described in both sections are not inhibitors of BubR1 and Bub3, respectively. Such claims are very misleading.
Section 7.0 "whereas BUB1B cooperates with multiple oncogenic regulators, including PLK1, MYC, CDC20, and Aurora kinases". Which Aurora kinases? Aurora A, Aurora B or Aurora C or a combination of them?
Figure 1 caption. "tetratricopeptide repeat (TPR) motifs essential for protein–protein interactions". In the context of Bub1 and BubR1 is mor appropriate to say the TPR mediates the kinetochore recruitment of both proteins. Also, as mentioned above, the description of BUB3 domain organisation is just wrong. Also,"BUBR1 contains two distinct KEN motifs and a similar S/T kinase domain, though kinase activity is considered pseudokinase in some contexts". What contexts exactly? This needs clarification.
Figure 6 caption. "...thereby preventing G2/M arrest or apoptosis" This statement needs further clarification. What drives each of these outcomes?
The acronym KBB is not listed in the abbreviations section.

Typo and Grammar issues:
Background section: "...responses, and autophagy positioning...."
3.4: "Consistently, abberant BUB1 and BUB1B expression..."
7.0: perhaps the authors meant potential off-target toxicities?
Ref 24 needs amendment.

One other concern is the inclusion of webpages of vendors in the list of references (ref 142, 147, 153). The primary research papers must be cited instead. For example, the original paper describing reversine as an Mps1 inhibitor was published by Santagida S et al J Cell Biol. 2010.

In summary, major corrections are required before this manuscript can be considered for its publication in Communications Biology.

Reviewer #2

(Remarks to the Author)
Overall, this is a nicely written review on BUB proteins. This article will improve with incorporation of few minor suggestions:

1. Include a section on BUB1 genes, their loci, genomic loss/overexpression and correlation with cancers and other disorders.
2. BUB1 regulates telomere length/stability. A section should be added on that.
3. Line 165 "phosphor-MELT"? a typo?
4. Line 600 "269,272,296,297"? Typo for references....copied/pasted from somewhere but not properly incorporated?
5. Section 7 feels like a stretch. There is a lot of redundant information. This section should be significantly reduced.

Version 2:

Reviewer comments:

Reviewer #1

(Remarks to the Author)
The authors have satisfactorily addressed the majority of issues I brought to their attention. One important aspect remains to be amended, though. The tetratricopeptide repeat motif is mentioned a couple of times in section 2.0 Evolution and Structural Organization, as a protein domain. In other instances such as Figure 1 caption, it is described as a protein motif. Please, be consistent with the use of nomenclature. The tetratricopeptide repeat is a protein motif and should be mentioned as such. It would be even better to say that the N-terminal region of Bub1 and Bub1B harbours a triple tandem of the tetratricopeptide repeat motif.
Once this issue has been amended, I would support the publication of this manuscript.

Reviewer #2

(Remarks to the Author)

Open Access This Peer Review File is licensed under a Creative Commons Attribution 4.0 International License, which permits use, sharing, adaptation, distribution and reproduction in any medium or format, as long as you give appropriate credit to the original author(s) and the source, provide a link to the Creative Commons license, and indicate if changes were made.
In cases where reviewers are anonymous, credit should be given to 'Anonymous Referee' and the source.
The images or other third party material in this Peer Review File are included in the article's Creative Commons license, unless indicated otherwise in a credit line to the material. If material is not included in the article's Creative Commons license and your intended use is not permitted by statutory regulation or exceeds the permitted use, you will need to obtain permission directly from the copyright holder.
To view a copy of this license, visit https://creativecommons.org/licenses/by/4.0/

Response to Reviewer 1 Comments

We sincerely thank Reviewer 1 for the careful and comprehensive evaluation of our manuscript. We greatly appreciate the insightful comments and constructive suggestions, which have substantially improved the scientific rigor, clarity, and overall presentation of the review. We have carefully considered each comment and have revised the manuscript accordingly. A detailed point-by-point response to all comments is provided below, and all corresponding changes have been incorporated into the revised manuscript.

Comment 1: BubR1 and BUB1B are used throughout the manuscript to refer to the same protein. For the sake of clarity, I recommend mentioning at the beginning of the manuscript that an alternative nomenclature exist (either BubR1 or BUB1B) and use only one of such names afterwards.
Response: We thank the reviewer for this helpful suggestion. We agree that consistent nomenclature improves readability. Accordingly, we have introduced the alternative nomenclature in the background section by stating that BUB1B is historically referred to as BubR1, and for consistency we have used BUB1B throughout the manuscript.

Comment 2: Section 2.0 Evolution and Structural Organization. Because a proper evolutionary analysis of BUBs requires a thorough comparison of BUBs orthologues and paralogues across species, I feel this topic distracts the attention from the main aim of this work. Namely, an in-depth analysis of the impact of human BUB proteins in oncogenic processes and their potential use in cancer therapeutics.
Response: We thank the reviewer for this insightful comment. While the primary focus of this review is the role of human BUB proteins in cancer, we believe that a brief overview of their evolutionary origin and structural organization provides important context for understanding the functional divergence of BUB1, BUB1B, and BUB3, which underpins their distinct biological and pathological roles discussed throughout the review.

Comment 3: The fact Figure 1 shows a wrong description of domains in Bub3, does not help to mitigate my concerns about the limited value of this section. Bub3 is a globular protein mostly organised as a 7-blade beta propeller rather than in two large regions of low structural complexity as the authors claim in this figure (admittedly, in the main text the authors refer to the WD40 fold when discussing Bub3 structure). There is nothing in Figure 1 that explains gene duplication events in the Bubs, really.

Response: We thank the reviewer for this valuable comment. In response, Figure 1 has been substantially revised. The incorrect representation of BUB3 has been replaced with its correct structural organization as seven WD40 repeats forming a seven-bladed β-propeller. Additionally, the evolutionary schematic has been redesigned to provide a clearer representation of the proposed gene duplication of the ancestral MadBub protein giving rise to the human BUB1 and BUB1B proteins, while maintaining the primary focus on the domain organization of the BUB family.

Comment 4: Figure 2 needs improvement. The caption lacks a proper description of what this figure intents to convey (for example, Sgo1 is shown in the two cartoons but it was not even mentioned in the figure legend). The MELT motifs of KNL1 appear near the C-terminal end when they should be shown in KNL1 N-terminal end.

Response: We thank the reviewer for this valuable suggestion. In response, Figure 2 has been extensively redesigned to specifically illustrate the checkpoint-unsatisfied state of the spindle assembly checkpoint. The figure legend has been substantially expanded to accurately describe all key molecular events and components depicted in the figure, including the roles of Sgo1, PP2A-B56, and cohesin protection. In addition, the schematic of KNL1 has been corrected by repositioning the MELT motifs to the N-terminal region, consistent with the established domain organization of KNL1.

Comment 5: Figure 3 shows essentially the same information shown in Figure 2: the series of events that drive a SAC response. The same can be said of Figure 6 Panel C.

Response: We appreciate the reviewer's constructive comment and agree that the previous Figures 2 and 3 contained overlapping information. Following the reviewer's suggestion and the editor's recommendation, the original figure has been reorganized

into two complementary figures with distinct biological focus. Figure 2 now exclusively illustrates spindle assembly checkpoint activation under checkpoint-unsatisfied conditions, whereas Figure 3 specifically depicts spindle assembly checkpoint silencing under checkpoint-satisfied conditions, including MCC disassembly, APC/C activation, cyclin B and securin degradation, separase activation, cohesin cleavage, and anaphase onset. This restructuring eliminates redundancy, improves clarity, and provides a clearer representation of the sequential molecular events governing SAC activation and silencing. In contrast, Figure 6C was retained because it serves a different purpose, illustrating the functional interplay between BUB proteins and the chromosomal passenger complex (CPC) in regulating kinetochore tension correction and APC/C inhibition within the context of oncogenic signalling, rather than depicting the canonical sequence of SAC activation and silencing.

Comment 6: Inhibitors targeting BubR1 and Bub3. This is misleading as none of the molecules described actually target BubR1 nor Bub3 directly. For instance, TAK-960 was described as an inhibitor of BubR1. However, this compound is a PLK1 inhibitor. Similarly, ZM447439 is an Aurora kinase inhibitor. Sections 6.2 and 6.3 must be rewritten completely or removed.

Response: We thank the reviewer for this important observation and agree that the original section headings could incorrectly imply that the discussed compounds directly inhibit BUB1B or BUB3. To address this, we have thoroughly revised Sections 6.2 and 6.3. The section headings have been changed to "Indirect Modulators of BUB1B-Associated Checkpoint Signalling" and "Indirect Modulators of BUB3-Associated Checkpoint Signalling", respectively. We have also added introductory statements explicitly noting that no selective direct pharmacological inhibitors of BUB1B or BUB3 have been reported to date.

Comment 7: For clarity, the list of abbreviations must be presented in alphabetical order. APC and APC/C are mentioned twice. The mention of the use of the APC acronym in a different context (and therefore meaning) is not relevant to this work

and should be removed. In the list of abbreviations each protein should be listed separately. For instance, interleukine 6 and interleukine 8 rather than IK6 and 8; Caspase-3 and Caspase-9.

Response: We thank the reviewer for this helpful suggestion. The abbreviation list has been thoroughly revised and reorganized in alphabetical order. Redundant entries have been removed, and grouped abbreviations have been separated into individual entries to improve clarity, consistency, and conformity with journal style.

Comment 8: Some acronyms are incorrect. For example Survivin Baculoviral IAP Repeat Containing 5 (BIRC5), AKT protein kinase B and p65.

Response: We thank the reviewer for this helpful suggestion. The abbreviation list has been revised to ensure accurate and standardized nomenclature. Survivin has been replaced with its official gene symbol, BIRC5 (Baculoviral IAP Repeat Containing 5), and the entries for AKT and p65 have been removed from the abbreviation list to avoid incorrect abbreviation expansion.

Comment 9: Some statement lack scientific rigour; A few examples are: "(BUBR1) C-terminal kinase domain primarily functions as a pseudokinase. More description of what this mean seems appropriate.

"Tumor protein p53" (p53 is a tumour supressor protein)

4.2: "Extending these findings, resistant organoid models..." Resistant to what exactly? This should be spelled out.

4.5: "Although BUB3 lacks catalytic activity, its elevated expression in immunologically “cold” tumors such as breast and lung cancers suggests potential involvement in immune exclusion and transcriptional adaptation" how exactly Bub3 may be involved in these processes? This needs clarification. Also "However, many current studies remain correlative" please mention specific examples here.

Sections 6.2 and 6.3 Again, the molecules described in both sections are not inhibitors of BubR1 and Bub3, respectively. Such claims are very misleading. Section 7.0 "whereas BUB1B cooperates with multiple oncogenic regulators, including

PLK1, MYC, CDC20, and Aurora kinases". Which Aurora kinases? Aurora A, Aurora B or Aurora C or a combination of them?

Response: We thank the reviewer for these valuable comments, which have improved the scientific rigor and clarity of the manuscript. Accordingly:

- The description of the BUB1B (BUBR1) pseudokinase domain has been revised to clarify that, although it lacks catalytic activity, it contributes to spindle assembly checkpoint signaling by serving as a structural scaffold for protein–protein interactions.
- "Tumor protein p53" was corrected to "tumor suppressor protein p53."
- Section 4.2 was revised by removing the unsupported reference to "*resistant organoid models*" and replacing it with an accurate description of patient-derived organoid models.
- Section 4.5 was revised to avoid unsupported mechanistic claims regarding BUB3, explicitly distinguish correlative findings from functional evidence, and conclude that further in vivo and mechanistic studies are needed to establish causal roles for BUB proteins in immune regulation.
- Sections 6.2 and 6.3 were revised to clarify that the discussed compounds target BUBR1- and BUB3-associated pathways, rather than acting as direct inhibitors.
- Section 7.0 was revised by removing the unsupported statement regarding BUB1B cooperation with Aurora kinases and distinguishing the experimentally supported functions of BUB1 and BUB1B.

Comment 10: Figure 1 caption. "tetratricopeptide repeat (TPR) motifs essential for protein–protein interactions". In the context of Bub1 and BubR1 is mor appropriate to say the TPR mediates the kinetochore recruitment of both proteins. Also, as mentioned above, the description of BUB3 domain organisation is just wrong. Also,"BUBR1 contains two distinct KEN motifs and a similar S/T kinase domain, though kinase activity is considered pseudokinase in some contexts". What contexts exactly? This needs clarification.

Response: We thank the reviewer for this helpful comment. Accordingly, we have revised both Figure 1 and its legend. The function of the TPR motifs has been corrected

to describe their role in mediating kinetochore recruitment through interaction with KNL1. The domain organization of BUB3 has been updated to accurately depict its seven WD40 repeats forming a seven-bladed β-propeller. In addition, the ambiguous statement regarding the BUB1B kinase domain has been clarified by describing it as a catalytically inactive pseudokinase domain. The figure has also been updated to provide a clearer representation of the proposed evolutionary origin of the BUB family through gene duplication.

Comment 11: Figure 6 caption. "...thereby preventing G2/M arrest or apoptosis" This statement needs further clarification. What drives each of these outcomes? The acronym KBB is not listed in the abbreviations section.

Response: We thank the reviewer for this valuable comment. The Figure 6 legend has been revised to clarify the distinct outcomes of BUB1B-mediated suppression of the DNA damage response. Specifically, we now indicate that suppression of the p53–p21/p27 axis bypasses the G2/M checkpoint, while persistent suppression of p53 signaling also impairs apoptosis under genotoxic stress. In addition, KBB (KNL1–BUB3–BUB1 complex) has been included in the abbreviations list.

Comment 12: Typo and Grammar issues:

Background section: "...responses, and autophagy positioning...."

3.4: "Consistently, abberant BUB1 and BUB1B expression..."

7.0: perhaps the authors meant potential off-target toxicities?

Ref 24 needs amendment.

Response: We thank the reviewer for carefully identifying these typographical and grammatical issues. The manuscript has been thoroughly proofread, and the suggested corrections have been incorporated. Specifically, the sentence in the Background section has been revised for improved grammatical clarity, "abberant" has been corrected to "aberrant" in Section 3.4, and the text in Section 7.0 has been carefully reviewed. We have retained the term "on-target toxicities", as the discussed toxicities arise from inhibition of BUB proteins in normal proliferative tissues where they are physiologically required. In addition, Reference 24 was re-examined and

confirmed to be accurate and appropriate for the cited statement.

Comment 13: One other concern is the inclusion of webpages of vendors in the list of references (ref 142, 147, 153). The primary research papers must be cited instead. For example, the original paper describing reversine as an Mps1 inhibitor was published by Santagida S et al J Cell Biol. 2010.

Response: We thank the reviewer for this valuable suggestion. We agree that primary research articles are more appropriate than vendor webpages for supporting scientific statements. Accordingly, the vendor website citations (References 142, 147, and 153) have been removed and replaced with the corresponding primary research articles wherever available. The reference list has been revised accordingly.

Response to Reviewer 2 Comments

We would like to express our sincere gratitude to Reviewer 2 for the thoughtful and constructive comments on our manuscript. We appreciate the careful assessment and insightful suggestions, which have helped us improve the scientific rigor, organization, and clarity of the review. We have carefully addressed each comment and revised the manuscript accordingly. Our point-by-point responses are presented below.

Comment 1: Include a section on BUB1 genes, their loci, genomic loss/overexpression and correlation with cancers and other disorders.

Response: We thank the reviewer for this valuable suggestion. Accordingly, we expanded the beginning of Section 2 (Evolution and Structural Organization) to include a concise overview of the human BUB gene family, including the chromosomal loci of BUB1, BUB1B, and BUB3, together with a brief discussion of BUB dysregulation through aberrant expression and altered gene dosage. The associations of BUB family members with cancers and other disorders are discussed throughout the subsequent sections of the review, and therefore were not repeated in a separate section.

Comment 2: BUB1 regulates telomere length/stability. A section should be added on that.

Response: We thank the reviewer for this valuable suggestion. Accordingly, we have expanded Section 4.2 to include the non-canonical role of BUB1 in telomere maintenance. Specifically, we discuss the recruitment of the BUB3–BUB1 complex to telomeres through TRF2, the BUB1-mediated phosphorylation of TRF1, and the resulting promotion of efficient telomere DNA replication and maintenance of telomere integrity. This addition addresses the emerging evidence supporting the involvement of BUB1 in telomere homeostasis and genome stability beyond its canonical spindle assembly checkpoint functions.

Comment 3: Line 165 "phosphor-MELT"? a typo?

Response: We thank the reviewer for identifying this typographical error. The term has been corrected to "phospho-MELT" to reflect the accepted terminology for phosphorylated MELT motifs on KNL1.

Comment 4: Line 600 "269,272,296,297"? Typo for references....copied/pasted from somewhere but not properly incorporated?

Response: We thank the reviewer for pointing out this error. The incorrect reference citations have been corrected, and the reference numbering has been updated accordingly.

Comment 5: Section 7 feels like a stretch. There is a lot of redundant information. This section should be significantly reduced.

Response: We thank the reviewer for this valuable suggestion. In response, Section 7 has been revised by removing redundant information and improving its overall conciseness and flow.

Response to reviewer comment

Comment: The tetratricopeptide repeat motif is referred to as a protein domain in Section 2.0, whereas elsewhere, including the Figure 1 caption, it is described as a protein motif. The nomenclature should be made consistent. It would also be preferable to state that the N-terminal region of BUB1 and BUB1B harbours a triple tandem of the tetratricopeptide repeat motif.

Response: We thank the reviewer for highlighting this nomenclature issue. We have revised Section 2.0 to consistently refer to the tetratricopeptide repeat as a **TPR motif** rather than a domain and clarified that the N-terminal regions of BUB1 and BUB1B harbour **three tandem TPR motifs**. The terminology has also been made consistent in the Figure 1 legend.
