# Supplementary material for: BUB signaling in cancer and molecular insights into targeted therapies
Source: Commun Biol. 2026 Oct 16;9:1335. doi: 10.1038/s42003-026-10976-5 (PMC13675223; doi:10.1038/s42003-026-10976-5)
Supplement: Supplementary file 2 [file 42003_2026_10976_MOESM2_ESM.pdf]

Title: BUB signaling in cancer and molecular insights into targeted therapies

Supplementary Table 1: Structural and Functional Overview of BUB Family Proteins

Protein	Structural Domains	Key Structural Features	Post-Translational Modifications	Canonical Role (SAC)	Non-Canonical Functions	Key Interactors
**BUB1**	Kinase, GLEBS	- Conserved N-terminal region with a kinetochore localization domain (often contains Tetratrico-Peptide Repeat (TPR) motifs). - Intermediate, non-conserved region required for BUB3 binding (contains GLEBS motif). - C-terminal serine/threonine kinase domain.	Phosphorylation, SUMOylation	Kinetochore recruitment, checkpoint activation	DDR signaling, immune modulation	PLK1, Aurora B
**BUB1B**	Pseudo-kinase, KEN box	Similar to BUB1 with conserved N-terminal kinetochore localization domain (TPR motifs). - Contains a conserved region for BUB3 binding (GLEBS motif). - C-terminal serine/threonine kinase domain.	Acetylation, Phosphorylation	APC/C inhibition, delay anaphase	CIN, enhancer hijacking	CDC20, FOXM1
**BUB3**	WD40-repeat	- "Donut-shaped" WD40 protein with a seven-blade beta-propeller structure. Acts as a scaffolding protein.	Ubiquitination	Scaffold for MCC	Immune evasion, epigenetic regulation	BUB1/BUB1B, MAD2

Supplementary Table 2: Common GO Enrichment Terms for BUB1, BUB1B, and BUB3

GO Category	GO Term	Description (Relevance to BUB1, BUB1B, BUB3)	Shared/Specific
Biological Process	mitotic cell cycle checkpoint	Essential for delaying anaphase until all chromosomes are properly attached to the mitotic spindle.	Shared (Core function of all three)
	spindle assembly checkpoint	A subset of the mitotic checkpoint, specifically monitoring kinetochore-microtubule attachments.	Shared (Core function of all three)
	chromosome segregation	The process by which chromosomes are accurately distributed to daughter cells during cell division.	Shared (All involved in ensuring proper segregation)
	cell division	The broader process of a cell dividing into two or more daughter cells.	Shared
	protein phosphorylation	BUB1 and BUB1B are kinases, actively phosphorylating other proteins to regulate the cell cycle.	Shared (BUB1, BUB1B as kinases; BUB3 as a binding partner)
	regulation of cell cycle	Overall control and coordination of events during the cell cycle.	Shared
	anaphase-promoting complex (APC/C) inhibition	Key mechanism by which the SAC, involving these proteins, delays anaphase onset.	Shared
	protein localization to kinetochore	The recruitment and precise positioning of checkpoint proteins at the kinetochore.	Shared
	apoptotic process	Dysregulation of BUBs can lead to apoptosis, and they can also be involved in	Shared (Particularly BUB1B in some contexts)

		sensing chromosome missegregation.	
Molecular Function	protein serine/threonine kinase activity	The catalytic activity of BUB1 and BUB1B, adding phosphate groups to serine or threonine residues.	Shared (BUB1, BUB1B)
	ATP binding	Necessary for the kinase activity of BUB1 and BUB1B.	Shared (BUB1, BUB1B)
	protein binding	All three proteins interact with a multitude of other proteins within the SAC and kinetochore.	Shared (All three, e.g., BUB1-BUB3 complex, BUB1B-CDC20)
	histone H2A kinase activity	Specific kinase activity of BUB1.	Specific (BUB1)
Cellular Component	kinetochore	The protein structure on chromatids where spindle microtubules attach; a primary site of action for all three.	Shared (Crucial for all three)
	condensed chromosome kinetochore	Refers to the kinetochore specifically on condensed chromosomes during mitosis.	Shared
	mitotic checkpoint complex	The multi-protein complex formed at the kinetochore to inhibit APC/C.	Shared (All are components)
	cytoplasm	Where these proteins are synthesized and can be found when not localized to specific structures.	Shared
	nucleoplasm	Can be found in the nucleus during certain phases or when interacting with nuclear components.	Shared (Context-dependent)
	bub1-bub3 complex	A specific complex formed between BUB1 and BUB3, crucial for SAC function.	Shared (BUB1, BUB3)

Supplementary Table 3: Signaling Mechanisms Involving BUB Family Proteins in Cancer Biology

BUB Protein	Signaling Pathway	Key Interacting Partners	Functional Role	Cancer Types/ Models	Oncogenic Outcome	Therapeutic Implications	References
**BUB1**	Spindle Assembly Checkpoint (SAC)	MAD2, CDC20, Aurora B	Ensures mitotic arrest under improper microtubule attachment	General (HeLa, A549, etc.)	Maintains chromosomal stability	Target for selective checkpoint disruption	[1]
**BUB1**	DNA Damage Response (DDR)	ATM, ATR, γH2AX, p53	Facilitates checkpoint activation during genotoxic stress	NSCLC, LUAD, HCC	Enhanced G2/M arrest and repair signaling	Combination with DDR inhibitors (e.g., ATRi)	[2–4]
**BUB1B**	p53/p21 Feedback Loop	p53, E2F1, PLK1	Suppresses senescence via dampening p21	LUAD, CRC	Promotes aneuploidy tolerance	Synthetic lethality with TP53 mutation	[5,6]
**BUB1B**	Immune Checkpoint Modulation	PD-L1, Tregs	Correlates with immune exclusion phenotype	TNBC, LUAD, RCC	Resistance to ICB therapy	Dual inhibition with PD-1/PD-L1 axis	[7,8]
**BUB1B**	Microtubule Stabilization	Aurora B, PLK1	Maintains kinetochore tension and SAC fidelity	High-CIN tumors	Mitotic override, CIN	Combinatorial targeting with AURKB/PLK1 inhibitors	[9–11]
**BUB1/BUB3**	ceRNA Regulation	MALAT1, miR-193a-3p	Post-transcriptional regulation	HCC	Sustained overexpression of BUBs	Antisense oligonucleotide therapy	[12]

Supplementary Table 4: Comprehensive list of BUB Inhibitors

S. No.	Inhibitor Name	Structure	Mode of Action	Clinical Status	Reference
**Inhibitors of BUB1**					
1.	BAY 1816032	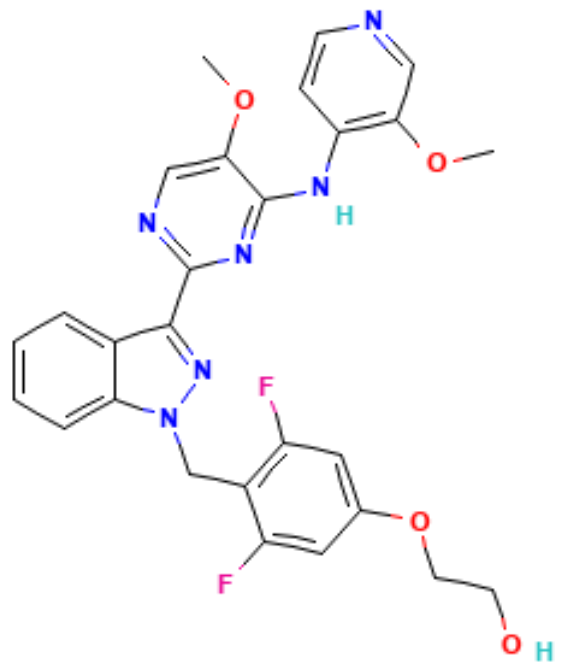	ATP-competitive kinase inhibitor; blocks BUB1 catalytic activity	Preclinical	PubChem CID: 118958833 [13]
2.	BAY-320	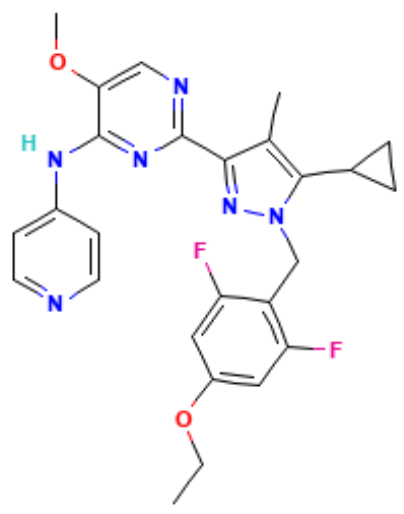	Small molecule kinase inhibitor; impairs SAC signaling and kinetochore checkpoint	Preclinical	PubChem CID: 71611462 [14]
3.	BAY-524	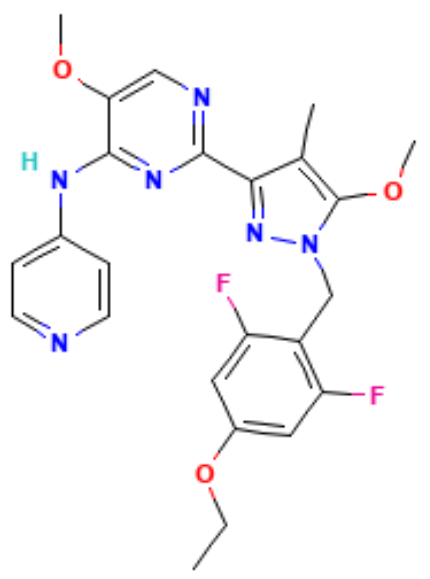	Highly selective inhibitor; blocks mitotic checkpoint signaling	Preclinical	PubChem CID: 71611179 [14]
4.	2OH-BNPP1	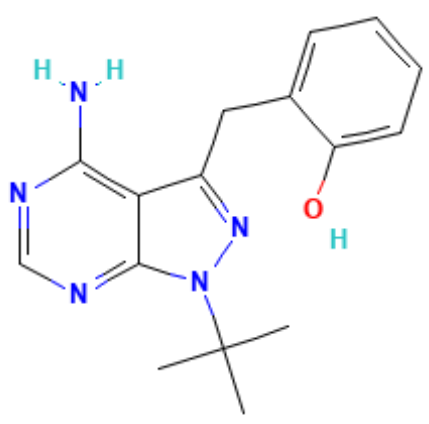	Specifically inhibits Bub1 and Bub1C and disrupts BUB	Preclinical	PubChem CID: 59796535 [15]

			checkpoint signaling		
**Inhibitors of BUB1B/BUBR1**					
1.	ZM447439	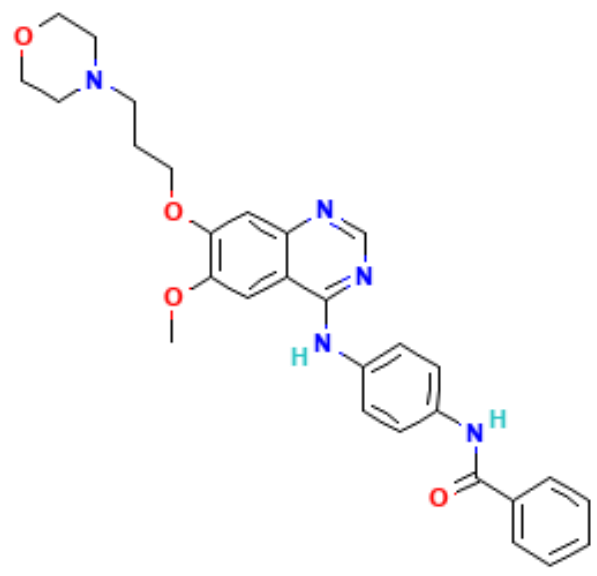	Inhibits Aurora B, disrupts SAC, indirectly affects BUB1/BUBR1 pathways	Preclinical	PubChem CID: 9914412 [16]
2.	TAK-960	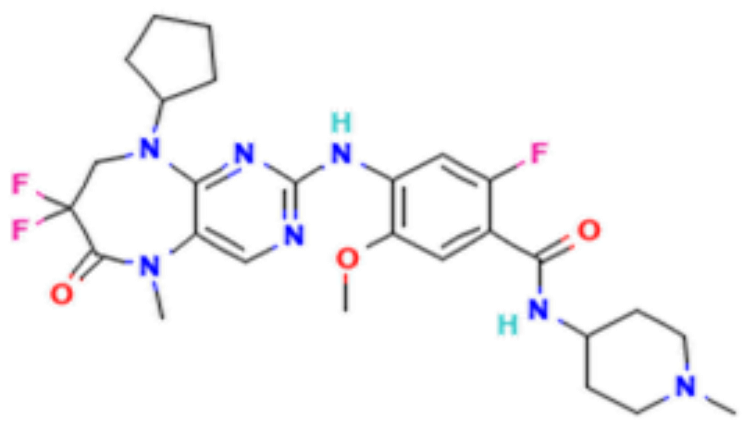	Aurora A kinase inhibitor; impairs mitotic spindle assembly, checkpoint via BUBR1	Phase I clinical trial (NCT01179399)	PubChem CID: 53357478 [17]
3.	Reversine	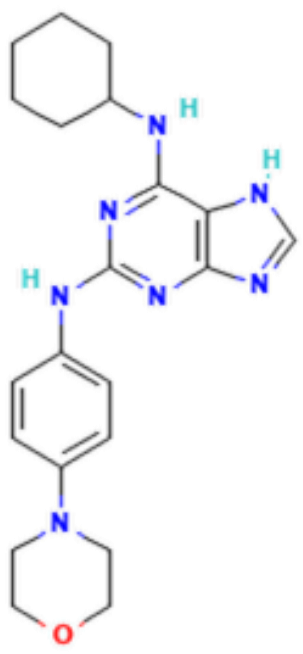	Aurora B kinase inhibitor; induces aneuploidy, affects BUBR1 signaling	Preclinical (cell line studies)	PubChem CID: 210332 [18]
**Inhibitors of BUB3**					

1.	GSK923295	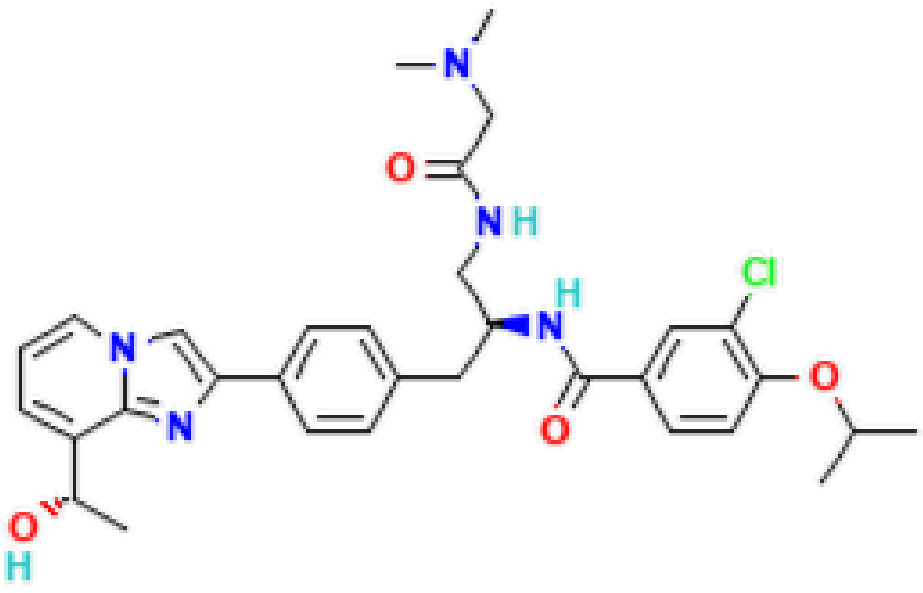	Indirectly interferes with BUB1-BUB3 interaction; impairs chromosome alignment	Phase I completed (NCT00573407)	PubChem CID: 46898058 [19]
**Inhibitors of BUB1 and BUBR1**					
1.	ZM447439	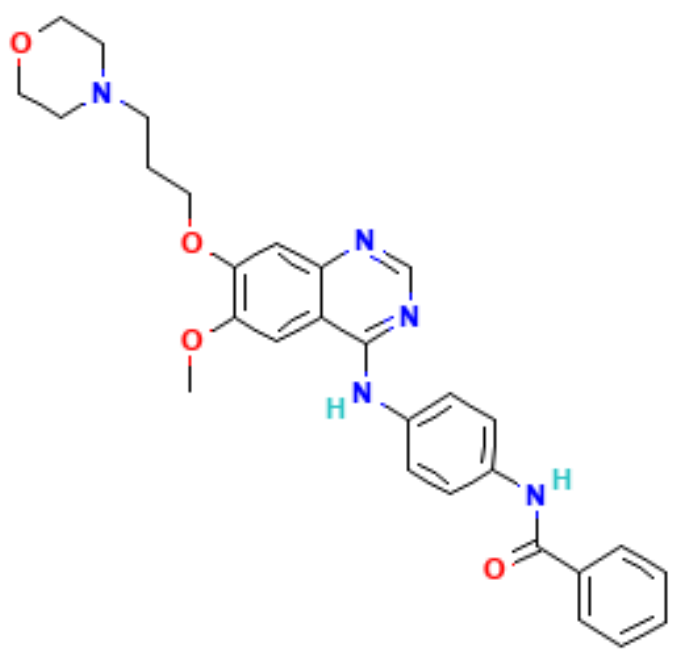	Inhibits Aurora B, disrupts SAC, indirectly affects BUB1/BUBR1 pathways	Preclinical	PubChem CID: 9914412 [20]

Supplementary Table 5: Clinical Correlates of BUB Family Gene Dysregulation Across Cancer Types

Cancer Type	Genes Involved	Clinical Impact	Reference
Triple-Negative Breast Cancer (TNBC)	BUB1	Correlates with poor prognosis, high Ki-67, and resistance to taxanes	21
Hepatocellular Carcinoma (HCC)	BUB1, BUB3	Associated with tumor grade, vascular invasion, and poor overall survival	22
Colorectal Cancer (CRC)	BUB1B	Linked to metastasis, CIN phenotype, and poor response to 5-FU chemotherapy	23
Prostate Cancer	BUB1	Predicts biochemical recurrence and castration resistance	24
Ovarian Serous Cystadenocarcinoma	BUB1, BUB1B	Associated with poor prognosis and enriched in BRCA1-mutated tumors	25
Lung Adenocarcinoma (LUAD)	BUB1, MAD2L1	Suggests a role in immune evasion; poor response to checkpoint inhibitors	26
Glioblastoma Multiforme (GBM)	BUB1B	Linked to tumor aggressiveness and radioresistance	27
Pancreatic Ductal Adenocarcinoma	BUB1	Supports tumor cell proliferation and predicts poor survival	28
Urothelial Bladder Cancer	BUB1B	Enriched in basal/squamous subtypes with immune exclusion signatures	29

Supplementary Table 6: Abbreviation List

Abbreviation	Full Form
ABBA	APC/C Binding and Activation Motif
ADP	Adenosine Diphosphate
AI	Artificial Intelligence
ALK5	Activin Receptor-Like Kinase 5
APC/C	Anaphase Promoting Complex/Cyclosome
ARCHS4	All RNA-seq and ChIP-seq Sample and Signature Search
ARID1A	AT-Rich Interaction Domain 1A
AST	Aspartate Aminotransferase
ATAC-seq	Assay for Transposase-Accessible Chromatin using Sequencing
ATP	Adenosine Triphosphate
ATR	Ataxia Telangiectasia and Rad3-related Protein
AUC	Area Under the Curve
AURKA	Aurora Kinase A
AURKB	Aurora Kinase B
BARD1	BRCA1-Associated RING Domain Protein 1
BAY	Bayer Compound
BCL-XL	B-Cell Lymphoma-Extra Large
BET	Bromodomain and Extra-Terminal Domain
BI 2536	Polo-Like Kinase 1 (PLK1) Inhibitor (from Boehringer Ingelheim)
BRCA1	Breast Cancer Type 1 Susceptibility Protein
BRD4	Bromodomain Containing Protein 4
BUB	Budding Uninhibited by Benzimidazole
BUB1	Budding Uninhibited by Benzimidazole 1
BUB1B	Budding Uninhibited by Benzimidazole 1 Beta
BUB3	Budding Uninhibited by Benzimidazole 3
BUBR1	Budding Uninhibited by Benzimidazole-Related Kinase 1
Caspase-3	Cysteine Aspartate Specific Proteases 3
Caspase-9	Cysteine Aspartate Specific Proteases 9
$CD8^+$ T cell	Cluster of Differentiation 8 Positive T Cell
CDC20	Cell Division Cycle Protein 20
CDK1	Cyclin-Dependent Kinase 1
CENP-E	Centromere Protein E
CENP-F	Centromere Protein F
ceRNA	Competing Endogenous RNA
cGAS–STING	Cyclic GMP-AMP Synthase–Stimulator of Interferon Genes
ChIP-seq	Chromatin Immunoprecipitation Sequencing
CHK1	Checkpoint Kinase 1
CHK2	Checkpoint Kinase 2
CIN	Chromosomal Instability
circRNA	Circular RNA

CLASP	Cytoplasmic Linker Associated Protein
CPC	Chromosomal Passenger Complex
CPTAC	Clinical Proteomic Tumor Analysis Consortium
CRISPR	Clustered Regularly Interspaced Short Palindromic Repeats
CRISPRa	CRISPR Activation
CRISPRi	CRISPR Interference
Cryo-EM	Cryo-electron Microscopy
CTLA-4	Cytotoxic T-Lymphocyte-Associated Antigen 4
DDR	DNA Damage Response
DNA	Deoxyribonucleic Acid
DSB	Double-Strand Break
E2F1	E2F Transcription Factor 1
EC_{50}	Half Maximal Effective Concentration
EMT	Epithelial–Mesenchymal Transition
ERK	Extracellular Signal-Regulated Kinase
FOXM1	Forkhead Box M1
G1/S	Gap 1 to Synthesis Phase Cell Cycle Transition
G2/M	Gap 2 to Mitosis Phase Cell Cycle Transition
GLEBS	GLE2-Binding Sequence
GO	Gene Ontology
GSCA	Gene Set Cancer Analysis
H2A-T120	Histone H2A Threonine 120
H3K27ac	Histone H3 Lysine 27 Acetylation
H3K4me3	Histone H3 Lysine 4 Trimethylation
HCC	Hepatocellular Carcinoma
HCP-1	Holocentric Chromosome Protein 1
HCP-2	Holocentric Chromosome Protein 2
HR	Homologous Recombination
I-BET762	Inhibitor of BET Bromodomains 762
IC_{50}	Half Maximal inhibitory concentration
ICB	Immune Checkpoint Blockade
IGF2BP1	Insulin-Like Growth Factor 2 mRNA-Binding Protein 1
IKK	IκB Kinase
IL-6	Interleukin 6
IL-8	Interleukin 8
IκBα	Inhibitor of Nuclear Factor Kappa B Alpha
JQ1	BET Bromodomain Inhibitor JQ1
KARD	Kinase Activity-Regulating Domain
KBB	KNL1–BUB3–BUB1 complex
KEN	Lysine-Glutamic Acid-Asparagine Motif
KNL1	Kinetochore Scaffold 1
KRAS	Kirsten Rat Sarcoma Viral Oncogene Homolog
LAG3	Lymphocyte Activation Gene 3
lncRNA	Long Non-Coding RNA

LUAD	Lung Adenocarcinoma
m6A	N6-Methyladenosine
MAD1	Mitotic Arrest Deficient 1
MAD2	Mitotic Arrest Deficient 2
MADBUB	Mitotic Arrest Deficient and BUB Kinase Ancestor
MALAT1	Metastasis-Associated Lung Adenocarcinoma Transcript 1
MAPK	Mitogen-Activated Protein Kinase
MCC	Mitotic Checkpoint Complex
MCL1	Myeloid Cell Leukemia 1
MDSCs	Myeloid-Derived Suppressor Cells
MELT	MPS1-Phosphorylated Motif in KNL1
MHC	Major Histocompatibility Complex
miRNA	MicroRNA
MPS1	Monopolar Spindle 1 Kinase
mRNA	Messenger RNA
MYC	MYC Proto-Oncogene
Ndc80	Nuclear Division Cycle 80 Homolog
NF-κB	Nuclear Factor Kappa-light-chain-enhancer of Activated B Cells
NHEJ	Non-Homologous End Joining
NSCLC	Non-Small Cell Lung Cancer
OTX015	BET Bromodomain Inhibitor OTX015
p53	Tumor suppressor Protein p53
PARP	Poly(ADP-ribose) polymerase
PCAF	p300/CBP-Associated Factor
PD-L1	Programmed Death-Ligand 1
PDX	Patient-Derived Xenograft
PI3K	Phosphoinositide 3-Kinase
PLK1	Polo-Like Kinase 1
PP2A-B56	Protein Phosphatase 2A, B56 Regulatory Subunit
PROTAC	Proteolysis Targeting Chimera
PTM	Post-Translational Modification
RB1	Retinoblastoma Protein
RNA	Ribonucleic Acid
ROD	Rough Deal Homolog
RZZ	ROD–ZW10–ZWILCH Complex
SAC	Spindle Assembly Checkpoint
SASP	Senescence-Associated Secretory Phenotype
SGO1	Shugoshin 1
SMARCA4	SWI/SNF Related, Matrix Associated, Actin Dependent Regulator of Chromatin, Subfamily A, Member 4
SNAI1	Snail Family Transcriptional Repressor 1
SSB	Single-Strand Break
BIRC5	Baculoviral IAP Repeat Containing 5 (Survivin)
TADs	Topologically Associating Domains

TCGA	The Cancer Genome Atlas
TF	Transcription Factor
TGFBR1	Transforming Growth Factor Beta Receptor 1
TGF-β/SMAD	Transforming Growth Factor Beta/SMAD Signaling Pathway
TIMER2.0	Tumor Immune Estimation Resource 2.0
TNBC	Triple-Negative Breast Cancer
TNF-α	Tumor Necrosis Factor Alpha
TPR	Tetratricopeptide Repeat
TRAF6	TNF Receptor-Associated Factor 6
Treg	Regulatory T Cell
TREX1	Three Prime Repair Exonuclease 1
VIM	Vimentin
WD40	Tryptophan-Aspartic Acid Repeat
WEE1	Wee1-Like Protein Kinase
Wnt/β-catenin	Wingless-related integration site/beta-catenin
YTHDF1	YTH N6-Methyladenosine RNA Binding Protein 1
ZEB1	Zinc Finger E-Box Binding Homeobox 1
ZNF143	Zinc Finger Protein 143
ZW10	Zeste White 10
ZWILCH	ZW10 Interacting Kinetochore Protein

References

1. Ando, J. *et al.* Changes in cell morphology and cytoskeletal organization are induced by human mitotic checkpoint gene, Bub1. *Biochemical and Biophysical Research Communications* **365**, 691–697 (2008).
2. Carrassa, L. & Damia, G. DNA damage response inhibitors: Mechanisms and potential applications in cancer therapy. *Cancer Treatment Reviews* **60**, 139–151 (2017).
3. Chen, R. *et al.* Budding uninhibited by benzimidazoles 1 overexpression is associated with poor prognosis and malignant phenotype: A promising therapeutic target for lung adenocarcinoma. *Thoracic Cancer* **14**, 893–912 (2023).
4. Zhang, L., Zhuge, Y. & Ni, J. BUB1 serves as a biomarker for poor prognosis in liver hepatocellular carcinoma. *BMC Immunology* **26**, 20 (2025).
5. Engeland, K. Cell cycle regulation: p53-p21-RB signaling. *Cell Death Differ* **29**, 946–960 (2022).
6. Zhao, Y. *et al.* Aberrations of BUBR1 and TP53 gene mutually associated with chromosomal instability in human colorectal cancer. *Anticancer Res* **34**, 5421–5427 (2014).
7. Sekino, Y. *et al.* BUB1B Overexpression Is an Independent Prognostic Marker and Associated with CD44, p53, and PD-L1 in Renal Cell Carcinoma. *Oncology* **99**, 240–250 (2021).
8. Zhang, H., Li, Y. & Lu, H. Correlation of BUB1 and BUB1B with the development and prognosis of endometrial cancer. *Sci Rep* **14**, 17084 (2024).

9. Chen, Q. *et al.* Bub1 and CENP-U redundantly recruit Plk1 to stabilize kinetochore-microtubule attachments and ensure accurate chromosome segregation. *Cell Reports* **36**, 109740 (2021).

10. Elowe, S., Hümmer, S., Uldschmid, A., Li, X. & Nigg, E. A. Tension-sensitive Plk1 phosphorylation on BubR1 regulates the stability of kinetochore–microtubule interactions. *Genes Dev* **21**, 2205–2219 (2007).

11. Song, C. *et al.* Self-priming of Plk1 binding to BubR1 ensures accurate mitotic progression. *Commun Biol* **7**, 1473 (2024).

12. Ma, Y., Shao, L., Shi, C. & Wang, J. Construction of Novel mRNA-miRNA-lncRNA Regulatory Networks Associated with Prognosis of HCV Related HCC. Preprint at https://doi.org/10.21203/rs.3.rs-951410/v1 (2021).

13. Siemeister, G. *et al.* Inhibition of BUB1 Kinase by BAY 1816032 Sensitizes Tumor Cells toward Taxanes, ATR, and PARP Inhibitors In Vitro and In Vivo. *Clin Cancer Res* **25**, 1404–1414 (2019).

14. Baron, A. P. *et al.* Probing the catalytic functions of Bub1 kinase using the small molecule inhibitors BAY-320 and BAY-524. *eLife* **5**, e12187 (2016).

15. The kinase activity of the Ser/Thr kinase BUB1 promotes TGF-β signaling | Science Signaling. https://www.science.org/doi/abs/10.1126/scisignal.2005379.

16. Marxer, M., Ma, H. T., Man, W. Y. & Poon, R. Y. C. p53 deficiency enhances mitotic arrest and slippage induced by pharmacological inhibition of Aurora kinases. *Oncogene* **33**, 3550–3560 (2014).

17. Dominguez-Brauer, C. *et al.* Targeting Mitosis in Cancer: Emerging Strategies. *Mol Cell* **60**, 524–536 (2015).

18. Huang, D. *et al.* Relation of AURKB over-expression to low survival rate in BCRA and reversine-modulated aurora B kinase in breast cancer cell lines. *Cancer Cell Int* **19**, 166 (2019).

19. Chung, V. *et al.* First-time-in-human study of GSK923295, a novel antimitotic inhibitor of centromere-associated protein E (CENP-E), in patients with refractory cancer. *Cancer Chemother Pharmacol* **69**, 733–741 (2012).

20. Ditchfield, C. *et al.* Aurora B couples chromosome alignment with anaphase by targeting BubR1, Mad2, and Cenp-E to kinetochores. *J Cell Biol* **161**, 267–280 (2003).

21. Zhou, R., Liu, M., Li, M., Peng, Y. & Zhang, X. BUB1 as a novel marker for predicting the immunotherapy efficacy and prognosis of breast cancer. *Transl Cancer Res* **13**, 4534–4554 (2024).

22. Saeki, A. *et al.* Frequent impairment of the spindle assembly checkpoint in hepatocellular carcinoma. *Cancer* **94**, 2047–2054 (2002).

23. De Angelis, P. M., Svendsrud, D. H., Kravik, K. L. & Stokke, T. Cellular response to 5-fluorouracil (5-FU) in 5-FU-resistant colon cancer cell lines during treatment and recovery. *Mol Cancer* **5**, 20 (2006).

24. Martinez, M. J., Lyles, R. D. Z., Peinetti, N., Grunfeld, A. M. & Burnstein, K. L. Inhibition of the serine/threonine kinase BUB1 reverses taxane resistance in prostate cancer. *iScience* **26**, (2023).

25. Qin, W. *et al.* Identification of key molecular markers in epithelial ovarian cancer by integrated bioinformatics analysis. *Taiwanese Journal of Obstetrics and Gynecology* **60**, 983–994 (2021).

26. Wang, Y. *et al.* Identification of key genes and biological pathways in lung adenocarcinoma via bioinformatics analysis. *Mol Cell Biochem* **476**, 931–939 (2021).
27. Lee, E. *et al.* Sensitivity to BUB1B Inhibition Defines an Alternative Classification of Glioblastoma. *Cancer Research* **77**, 5518–5529 (2017).
28. Li, F. *et al.* Positive feedback regulation between glycolysis and histone lactylation drives oncogenesis in pancreatic ductal adenocarcinoma. *Mol Cancer* **23**, 90 (2024).
29. Yamamoto, Y. *et al.* Overexpression of BUBR1 is associated with chromosomal instability in bladder cancer. *Cancer Genetics and Cytogenetics* **174**, 42–47 (2007).
